# Structural and functional analysis of the nucleotide and DNA binding activities of the human PIF1 helicase

**DOI:** 10.1093/nar/gkz028

**Published:** 2019-01-30

**Authors:** Saba Dehghani-Tafti, Vladimir Levdikov, Alfred A Antson, Ben Bax, Cyril M Sanders

**Affiliations:** 1 Department of Oncology and Metabolism, Academic Unit of Molecular Oncology, University of Sheffield, Beech Hill Rd., Sheffield S10 2RX, UK; 2 York Structural Biology Laboratory, Department of Chemistry, University of York, York YO10 5DD, UK; 3 Medicines Discovery Institute, Cardiff University, Main Building, Park Place, Cardiff CF10 3AT, UK

## Abstract

Pif1 is a multifunctional helicase and DNA processing enzyme that has roles in genome stability. The enzyme is conserved in eukaryotes and also found in some prokaryotes. The functions of human PIF1 (hPIF1) are also critical for survival of certain tumour cell lines during replication stress, making it an important target for cancer therapy. Crystal structures of hPIF1 presented here explore structural events along the chemical reaction coordinate of ATP hydrolysis at an unprecedented level of detail. The structures for the apo as well as the ground and transition states reveal conformational adjustments in defined protein segments that can trigger larger domain movements required for helicase action. Comparisons with the structures of yeast and bacterial Pif1 reveal a conserved ssDNA binding channel in hPIF1 that we show is critical for single-stranded DNA binding during unwinding, but not the binding of G quadruplex DNA. Mutational analysis suggests that while the ssDNA-binding channel is important for helicase activity, it is not used in DNA annealing. Structural differences, in particular in the DNA strand separation wedge region, highlight significant evolutionary divergence of the human PIF1 protein from bacterial and yeast orthologues.

## INTRODUCTION

Accurate DNA replication requires a suite of enzymes including helicases that translocate on DNA. Helicases can catalyse protein displacement from DNA (1) but they are known primarily for their ability to remodel DNA secondary structure (2) and generate single-stranded DNA (ssDNA) during DNA replication, repair, recombination or restart (3). Most replication helicases are modular enzymes. In addition to a catalytic ‘helicase core’ auxiliary domains may provide additional enzymatic functions required for DNA processing, such as nuclease activity (4) and DNA strand annealing functions (5), or a substrate targeting activity via a structure-specific DNA binding domain (6).

The founding member of the Pif1 protein family was identified in genetic screens in *Saccharomyces cerevisiae* as a gene involved in mitochondrial DNA recombination and stability (7). Later, the purified yeast protein, ScPif1, was shown to be a helicase (8) and nuclear DNA replication functions were also identified (9). In fission yeast it was demonstrated that the enzyme was required for the completion of S phase (10). Nuclear ScPif1 has roles in Okazaki fragment maturation (11,12), telomere length regulation (13), replication through loci that normally impede the replication fork (e.g. the rRNA Replication Fork Barrier, 14,15) and the resolution of G4 DNA structures (16,17). Purified ScPif1 is a DNA-dependent ATP*ase* and 5′–3′ helicase (8,18) that unwinds forked dsDNA substrates with ssDNA tails and RNA-DNA hybrids (19) and binds and unwinds G4 DNA (20). Like a subset of helicases, including RecQs (5,21), ScPif1 has a DNA strand annealing activity (22). Genome analysis has since identified at least one *Pif1*-like gene in almost all eukaryotes and, curiously, also some prokaryotes (23,24).

Pif1 proteins are monomeric enzymes and members of helicase superfamily 1 (SF1), while the 5′–3′ polarity of unwinding place them in the SF1B subgroup. Seven conserved amino acid motifs (I, Ia, II, III, IV, V and VI) have been identified in SF1 helicases that are located in two structurally related RecA-like domains, 1A and 2A. The RecA domains and two additional domains, 1B and 2B, form the helicase core (2). The nucleotide triphosphate (NTP) binding site required for helicase activity is structured by residues from both RecA domains. Here, motifs I (Walker A), II (Walker B) and IV are directly involved in NTP/Mg^2+^ binding and hydrolysis while the other conserved motifs (Supplementary Figure S1) are proposed to be involved in the energy transduction events coupling NTP*ase* to helicase activity (25). Pif1 also belongs to the RecD helicase sub-family that share three additional motifs, A in domain 2A and B and C in domain 2B (23). The function of these motifs has become apparent recently from studies of bacterial Pif1 proteins (26,27) and will be discussed further in this manuscript.

Although the overall biochemical activities of human PIF1 (hPIF1) are conserved relative to ScPif1 (28–30) its cellular functions are unclear. Nuclear and mitochondrial splice variants of hPIF1 exist (31) and the gene is not essential, as in *S. cerevisiae* (32). However, siRNA mediated depletion of the enzyme results in a delayed S-phase, indicating a role in the completion of DNA replication (31). Importantly, several studies indicated that hPIF1 may be required for the maintenance of replication fork progression during tumourigenesis, especially during replication stress induced by genotoxic drugs, including those used in cancer chemotherapy (33,34). hPIF1 has therefore been proposed as a cancer therapy target.

Here, we focus on hPIF1 for which little data are available due to challenges posed in producing protein suitable for structural and biochemical characterisation. The full-length nuclear form of hPIF1 is 641 amino acids, with the ∼45 kDa helicase core (hPIF1HD) residing in residues 206–620 (29). The functions of the segments N- and C-terminal of hPIF1HD are unclear, but the N-terminal residues 1-205 have a non-specific DNA binding activity that augments the activity of helicase core (28,30). Only a low-resolution (3.6 Å) structure of hPIF1HD is available and crystallization of hPIF1 with DNA has thus far failed (26,27), so structural studies initially focused on the more amenable *Bacteroides* spp. Pif1 (BsPif1) protein. Interestingly, the full-length 433 amino acid BsPif1 is structurally similar to hPIF1HD (26) although the proteins share only 21% sequence identity (Supplementary Figure S1).

Recent structures of Bs (26,27) and ScPif1 (35), with and without nucleotide cofactors and ssDNA bound have advanced understanding of the helicase mechanism. However, the chemo-mechanical chain of events in NTP hydrolysis and DNA unwinding remain poorly defined, as does the structural basis for Pif1's G4 DNA binding and strand annealing activities. Given the low level of sequence identity between microbial Pif1 and human PIF1 proteins it is also unclear whether these orthologues provide accurate templates for understanding hPIF1 functions. Here, we describe the first high-resolution crystal structures of hPIF1HD, including an apo structure at 1.44 Å, and a complex with the nucleotide analogue AMP-PNP at 1.13 Å. We also determined a structure with ADP and AlF_4_^−^ bound, mimicking a transition state intermediate. These structures allowed us to model the structural events along the chemical reaction coordinates of ATP hydrolysis and how they may be linked to helicase activity. This and the crystal structures of BsPif1 (26,27) and the ScPif1 helicase domain (residues 237–780) bound to ssDNA (35), provided the basis for a model of the ssDNA bound conformation of hPIF1. We probed possible modes of DNA binding and unwinding by site-directed mutagenesis and biochemical analysis of variant proteins. We conclude that while hPIF1 is likely to function generally as an ATP-dependent motor protein like the previously characterized ScPif1 and BsPif1, the functional sites for DNA binding and unwinding have diverged in sequence and activity.

## MATERIALS AND METHODS

### Protein expression and purification

Human PIF1 helicase domain proteins were expressed codon-optimized using pET system plasmids (pET15b) in ArcticExpress™ BL21(DE3) cells (Agilent Technologies) at 6°C for 3 days. hPIF1HD (residues 206–621) was expressed with an N-terminal hexa-histidine and thrombin cleavage site tag (His-tag) and hPIF1HD_206-END_ (residues 206–641) was expressed with an N-terminal hexa-histidine-maltose binding protein (His-MBP) tag, followed by a thrombin cleavage site. All purification steps were at 4°C. For both constructs, cells were lysed by sonication in 50 mM Tris–Cl pH 8.0 (4°C), 550 mM NaCl, 5 mM DTT, 10% (v/v) glycerol, 1 mM PMSF (3 ml/g of cells) and centrifuged at 25 000 × *g* for 30 min at 4°C. The NaCl concentration of the cleared lysate was adjusted to ∼1 M and nucleic acids removed by polyethylenimine P (5% w/v) precipitation. 0.226 grams of ammonium sulphate was added per ml of cleared solution and protein precipitated by centrifugation at 25 000 × *g* for 20 min at 4°C. The pellet was re-suspended in 50 mM Tris–Cl pH 8.0 (4°C), 500 mM NaCl, 2.5 mM DTT, 20 mM imidazole, 10% (v/v) glycerol and applied to a HisTrap Ni-Sepharose column (GE Healthcare) and eluted in a gradient developed to 200 mM imidazole over 15 column volumes (CV). The His or His-MBP tags were removed by thrombin cleavage, leaving four residues of the tag, GSRM.

hPIF1HD was further purified with a second round of Ni-sepharose chromatography, size exclusion chromatography (SEC, Superdex 200; 20 mM Tris–Cl pH 8.0 (4°C), 300 mM NaCl, 2.5 mM DTT, 5% (v/v) glycerol, 1 mM EDTA, 1 mM PMSF) and cation exchange chromatography (Source S; 20 mM NaPhosphate pH 7.2, 0.05 mM EDTA, 10% (v/v) glycerol, 2.5 mM DTT, 0.1 mM PMSF, gradient from 100–350 mM NaCl, 15 CV) and finally dialysed against 10 mM Tris–Cl pH 8.0, 200 mM NaCl, 2% (v/v) glycerol, 5 mM DTT, 0.1 mM PMSF, 0.1 mM EDTA.

hPIF1HD_206–641_ was further purified by SEC (as above), followed by a second round of Ni-sepharose chromatography and cation exchange chromatography (buffer as above, 50–300 mM NaCl gradient, 15 CV) and finally dialysed against 10 mM Tris–Cl pH 8 (4°C), 300 mM NaCl, 2% (v/v) glycerol, 5 mM DTT, 0.1 mM PMSF, 0.1 mM EDTA.

Proteins were concentrated to ∼30 mg ml^−1^ for storage (−80°C). Concentrations were determined from absorbance at 280 nm readings determined in 7M guanidinium hydrochloride using the calculated molar extinction coefficients.

### Crystallization and structure determination

Initial crystallization conditions were identified at 4°C from screens using 150 nl of precipitant solution and 150 nl of hPIF1 protein (10–15 mg ml^−1^). Crystals of the hPIF1HD-AMP-PNP complex were grown in hanging drops (4°C) by mixing the protein at a final concentration of 11 mg ml^−1^ in 10 mM Tris–Cl pH 8.0, 2% (v/v) glycerol, 5 mM MgCl_2_, 5 mM AMP-PNP, 2.5 mM TCEP, 0.3 M NaCl with a reservoir solution in a 1:1 ratio. The well solution contained 0.3 M Na-acetate, 17–27% (w/v) PEG 2K MME in 0.1 M Tris–Cl pH 7.5. For phasing, crystals were soaked briefly in mother liquid supplemented with 0.8 M KBr. All diffraction data were collected at cryo-conditions using synchrotron radiation at Diamond Light Source (DLS, Oxford) and processed with XDS (36). Data sets at three different wavelengths for Br-containing crystals (Supplementary Table S1) were collected for structure determination by the multiple-wavelength anomalous dispersion (MAD) method using SHELX (37). Crystals belonged to the *P*2_1_2_1_2_1_ space group and contained two molecules in the asymmetric unit.

Co-crystals of hPIF1HD-AMP-PNP in a different space group, C222_1_, diffracting to 1.13 Å resolution and containing one molecule per asymmetric unit, were grown using 11 mg ml^−1^ protein solution in 0.1 M Tris–Cl pH 7.5, 1% (v/v) glycerol, 5 mM MgCl_2_, 5 mM AMP-PNP, 2.5 mM TCEP, 0.3 M NaCl, 0.3 M Na-acetate, 10% (w/v) PEG 8K and 10% (w/v) PEG 1K. The apo structure of hPIF1HD_206–641_ was obtained from crystals belonging to the *P*2_1_2_1_2_1_ space group with one protein molecule per asymmetric unit grown using 15 mg ml^−1^ protein solution in 0.1 M MES pH, 6.0, 0.5% (v/v) glycerol, 5 mM MgCl_2_, 2.5 mM TCEP, 150 mM NaCl 0.2 M Li_2_SO_4_ and 25% (w/v) PEG 2KMME at 4°C. The hPIF1HD-ADP•AlF_4_^−^ co-crystals in the *P*3_1_21 space group with two molecules per asymmetric unit were grown using 13 mg ml^−1^ protein solution in 0.1 M MES pH 6, 1% (v/v) glycerol, 5 mM MgCl_2_, 5 mM ADP, 6 mM AlCl_3_, 50 mM NaF, 150 mM NaCl, 2.5 mM TCEP, 0.2 M Ca-acetate, 8% (w/v) PEG 20K and 8% (w/v) PEG 550MME. The coordinates of the refined Br derivative structure were used as a molecular replacement search model using the data obtained from the hPIF1HD-AMP-PNP, apo hPIFHD_206–641_ and ADP•AlF_4_^−^-hPIF1HD crystals. An unambiguous solution was found using MOLREP (38). All atomic models were built with COOT (39) and refined using a restrained maximum likelihood approach implemented in REFMAC (40), Supplementary Table S1.

### Modelling of the human PIF1 helicase core complex with ssDNA bound

An initial model of a ssDNA bound conformation of hPIF1HD was made by superposing domains from the 1.13 Å human complex with AMP-PNP onto the equivalent domains in the 2.0 Å BsPif1 complex with ssDNA and ADP•AlF_4_^−^ (pdb code: 5FHD), and using the ssDNA coordinates from 5FHD. The human PIFHD-ssDNA model was then energy minimized in Maestro (41). A second model of the ssDNA bound conformation of human PIF1HD was made when coordinates for the more closely related ScPif1 became available (the 2.03Å structure of ScPif1 in complex with GGGTTT and ADP•AlF_4_^−^ (35), PDB code: 5O6B).

### DNA binding assays

Single stranded DNA binding reactions were performed with a poly-T_(35)_ substrate (0.4 nM), end-labelled with ^32^P using polynucleotide kinase (pnk) and [γ-^32^P]ATP (6000 Ci/mmol) and purified from poly-acrylamide gels as described previously (29). The binding buffer was 20 mM HEPES–NaOH pH 7.5, 135 mM NaCl, 5% (v/v) glycerol, 1 mM DTT and 1 mg ml^−1^ BSA. Reactions were incubated for 20 min at 20°C before resolving complexes on 5% poly-acrylamide gels (29:1) using 0.25× TBE running buffer. The radiolabelled tetramolecular G4 DNA substrate used was formed from the single-stranded precursor 5′-TTTTTTTTTTGGGGTTTTGGGG as described previously (30). The reaction buffer (0.1 nM G4 DNA) was as described above, except glycerol was omitted from the reactions and replaced with 2% (w/v) PEG 8000 and the reactions contained 5 μM poly-T_(35)_ competitor ssDNA. Reaction products were visualized and quantified following exposure of the dried gels to phosphorimaging plates. Data analysis was performed using the program PRISM (GraphPad), as were all other biochemical data described below.

### Helicase assays

A radiolabelled partially single- and double-stranded test substrate was generated by annealing the following oligonucleotides: 5′-(T)_55_-CGAATTCGAGCTCGGTACCC and 5′-GGGTACCGAGCTCGAATTCG, as described previously (29). The reaction buffer was 20 mM HEPES–NaOH pH 7.5, 20 mM NaCl, 5 mM MgCl_2_, 2 mM ATP, 1 mM DTT and 0.1% (v/v) NP-40. Reactions (0.1 nM substrate) were incubated at 20°C for 30 min and terminated by the addition of 0.2 volumes of 120 mM EDTA, 0.6% (w/v) SDS, 60% (v/v) glycerol and 0.1% (w/v) bromophenol blue before polyacrylamide gel electrophoresis (8% (19:1), 0.25× TBE/0.05% (w/v) SDS) and exposure of dried gels to phosphorimaging plates.

### ATP*ase* assays

A charcoal binding assay (42) was used to measure DNA-dependent ATP hydrolysis for 100 nM hPIF1HD/200 nM poly-T_(30)_ as described previously (29). The reaction buffer used was 20 mM HEPES–NaOH pH 7.5, 75 mM NaCl, 5 mM MgCl_2_, 5 mM ATP, 0.0125 mM [γ-^32^P]ATP (6000 Ci/mmol), 1 mM DTT, 0.1 mg ml^−1^ BSA and 0.1% (v/v) NP-40. Phosphate release was determined after 10 min (20°C) when ∼3% of the ATP was hydrolysed.

### DNA strand annealing assay

Strand annealing assays employed two radiolabelled oligonucleotides with a complementary sequence of 20 bases and non-complementary 55 nucleotide 5′ or 30 nucleotide 3′ overhangs-5′-(T)_55_-CGAATTCGAGCTCGGTACCC and 5′-GGGTACCGAGCTCGAATTCG-(C)_30_. The reaction buffer was 20 mM HEPES–NaOH pH 7.5, 135 mM NaCl, 1 mM DTT and 0.1% (v/v) NP-40. The annealed substrate was gel-purified and heat denatured before addition to the reaction (0.1 nM final concentration). Reactions were incubated and processed as described for the helicase assays.

### Preparation of variant hPIF1 proteins

Variants of hPIF1HD (residues 206–620) were made by site directed mutagenesis by primer extension and purified as described above. Protein concentrations of mutant and wild-type proteins for biochemical assays were determined using the Bradford assay (BioRad) from triplicate measurements performed in parallel using BSA as a standard.

## RESULTS

### hPIF1 structure determination and overview

**Figure 1.**
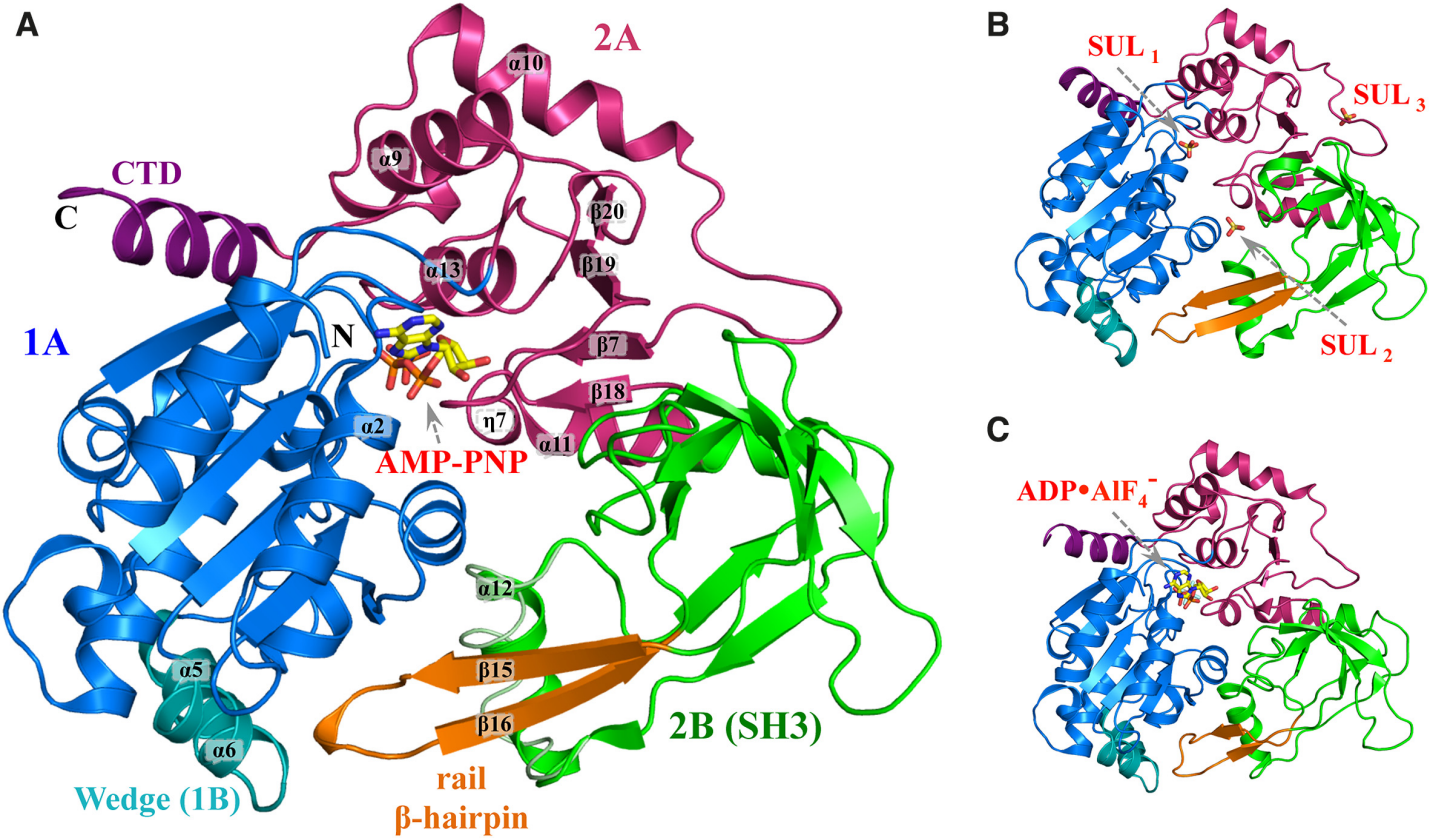
Structural overview of hPIF1. Cartoon representation of the hPIF1 helicase core domain. (**A**) The 1.13 Å hPIF1-AMP-PNP structure. Domains are coloured as 1A (blue), 2A (red) and 2B (green). Note the C-terminal domain (CTD, purple) and the functional wedge domain (1B, cyan) are structurally part of domain 1A. The rail β-hairpin (orange) is part of domain 2B (a SH3 domain). Secondary structure elements α2, α5, α6, α9, α10, α11, α12, α13, β7, β15, β16, β18, β19, β20, η7 are labeled. An alternative conformation of the α12 helix is shown in tube representation. (**B**) and (**C**), the 1.44Å hPIF1-apo structure with the position of the three bound sulphates (SUL_1–3_) indicated and the low-resolution hPIF1-ADP•AlF_4_^−^ structure respectively. The domains are coloured as in (A).

High resolution X-ray structures were determined for the helicase domain of hPIF1 (Figure 1) corresponding to an idle cycle of ATP hydrolysis: structures of complexes with the ground state analogue AMP-PNP in two different crystal forms (1.13 Å, 1.43 Å; Figure 1A), an apo structure complexed with three sulphate moieties determined at 1.44Å resolution (Figure 1B) and also a low-resolution structure of a complex with the transition state analogue ADP•AlF_4_^−^, Figure 1C (see Supplementary Table S1 for details of the data collection and refinement statistics). While the hPIF1-ADP•AlF_4_^−^ structure (*R*_factor_ 17.9%; *R*_free_ = 25.3%) mirrors a previously published low resolution (3.6 Å) crystal structure of the hPIF1 helicase domain (residues 200–641, pdb code: 5FHH, *R*_factor_ 31.3%; *R*_free_ = 35.5%, (26)), it is based on using the high-resolution structure of the hPIF1-AMP-PNP complex during molecular replacement, resulting in a more accurate model than the previously available structure (RMS_CA_ = 2.0 Å; RMS_overall_ = 2.5 Å). Notably, average temperature factors (Supplementary Table S1) indicate significant flexibility of the hPIF1-ADP•AlF_4_^−^ structure relative to all others. The structures we report are of constructs from residues 206–641 (apo hPIF1) or 206–620 (the structures with nucleotide analogues bound). In the 1.44 Å apo-hPIF1 crystal structure residues after 620 are not seen in the electron density suggesting they are disordered. Our two constructs (which are henceforth called hPIF1) are active in helicase/DNA binding assays and demonstrate characteristic DNA dependent ATP*ase* activity.

**Figure 2.**
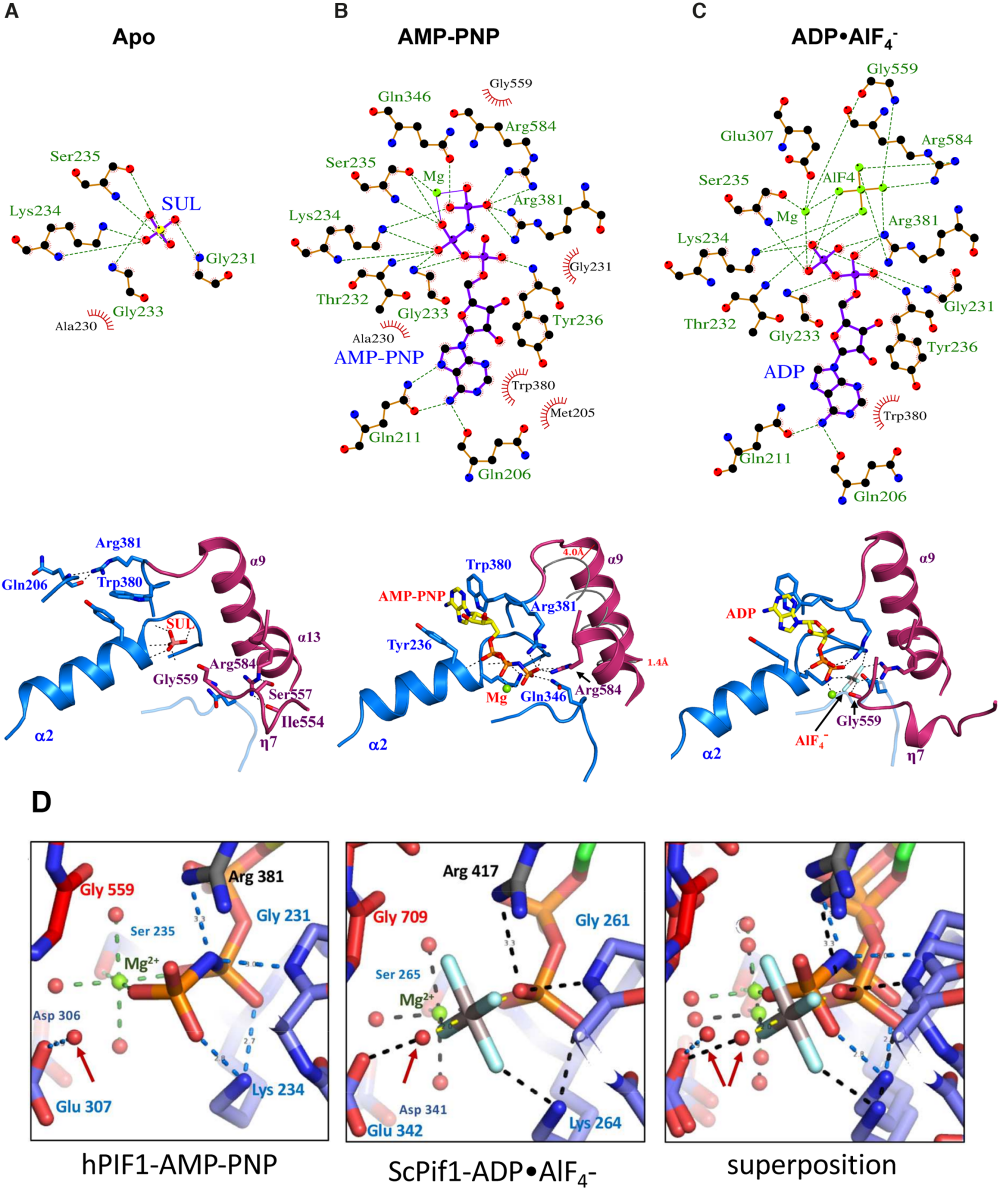
Nucleotide binding and conformational changes in hPIF1. (**A**) LIGPLOT (45) scheme (above) and structural representation of critical interactions (below) at the nucleotide binding site of apo hPIF. The green dashed lines represent hydrogen bonds and the non-ligand residues involved in hydrophobic contacts are indicated with annotations in red. Note, the sulphate ion is coordinated at the position of the ATP β-phosphate. (**B**) and (**C**), as in (A) for the AMP-PNP and ADP•AlF_4_^−^ bound hPIF1 structures. In (B), centre, the conformation of the α9 helix in apo hPIF1 is represented by a grey tube and the relative displacement is indicated. (**D**) A comparison of the ATP binding site in hPIF1-AMP-PNP (left), ScPif1-ADP•AlF_4_^−^ (centre) and the superposition of the two (right). The attacking water is indicated with an arrow in each case.

The hPIF1 helicase has the domain architecture characteristic of the SF1B RecD2 helicase subgroup (2,43). The overall organization of these domains is similar in all nucleotide bound and free structures determined (Figure 1A–C). hPIF1 has three structural domains, two RecA-like domains 1A (residues 206–381, blue in Figure 1) and 2A (residues 382–435, red in Figure 1) and domain 2B (residues 436–548, green in Figure 1) with a SRC Homology 3 (SH3)-like fold, in addition to the small functional domain 1B within domain 1A (residues 280–300, cyan in Figure 1). The ATP binding site is located between domains 1A and 2A. A notable feature of domain 2B is a rail-like β-hairpin (orange in Figure 1) with its tip proximal to domain 1B. A small C-terminal domain (CTD, purple in Figure 1), protruding from domain 2A contains an α-helix which packs against and is structurally part of domain 1A. In addition, the α11 helix (residues 429–442) is shared between domains 2A and 2B. The side-chain of the conserved N436, which is in the middle of the α11 helix, makes two hydrogen bonds to the main-chain NH and CO of A551 in domain 2B, keeping the C-terminus of the α11-helix relatively fixed (and part of) the SH3 domain. The domain boundaries and secondary structure names we have used here broadly follow those for the yeast and *Bacteroides* Pif1 structures (26,27,35). However, we note that hPIF1 and BsPif1 do not contain the additional 2C domain inserted in the 2B domain of ScPif1 (see Supplementary Figure S1).

The functional domain 1B contains the α5 and α6 helices and is stabilized by interactions with the α-helical Pif1 family signature motif (PFSM, refs. 26,27). In ScPif1 there are also two α-helices but in BsPif1 there is no corresponding helix at the α5 position. The 1B ‘wedge’ domain is believed to play a role in separating the incoming DNA duplex. In other SF1B helicases a corresponding β-hairpin serves the same role (43,44). Importantly, the amino acid sequence of the wedge is not conserved. In ScPif1 the sequence KKVRRSRKHLRR at the C-terminal end of helix α5 and the N-terminal end of helix α6 contains many positively charged residues. The equivalent sequence in hPIF1 is very different (ALAQ-RPGVRQG), and the sequence is again different in BsPif1 (ENK-FSEYKVEL).

### ATP binding and hydrolysis

ATP binding and hydrolysis are accompanied by significant rearrangement in the hydrogen-bonding network, not only in the ligand binding site (Figure 2) but also throughout the whole protein structure, particularly at interdomain interfaces. Only key interactions will by described further below, although all interactions are detailed using a LIGPLOT (45), Figure 2A–C.

In the apo-hPIF1 structure the position of the β-phosphate of ATP is occupied by a sulphate moiety, SUL_1_ in Figure 1. SUL_1_ is stabilized by hydrogen bonds with the main chain atoms of the loop residues from the Walker A motif (Supplementary Figure S1) and the side chain of the Walker A K234 residue (Figure 2A) at the beginning of the α2 helix, situated at the 1A–2A inter-domain contact area. Tight packing of this area is maintained by interaction of the inter-domain linker R381 (motif IV) with the carboxyl group of Q206 at the N-terminal part of the 1A domain. A second arginine, R584 (motif VI), interacts with S557 (motif V) and the carboxyl group of I554, contributing to the rigidity of the inter-domain 2A–2B contact area (Figure 2A).

During ATP binding, represented by the AMP-PNP structure, R381 recognizes the γ-phosphate of ATP (Figure 2B). This interaction involves a significant movement of the residue and the 1A–2A inter-domain linker relative to the apo conformation. As such, the aromatic side chain of the adjacent W380 (motif IV) then stacks with the adenine base and together with Y236 forms a pocket for the base. R584 also shifts in position to interact with the ATP γ-phosphate, fixing the α13 helix (domain 2A) relative to the ATP-binding pocket, while Q346 from motif III coordinates the ATP γ-phosphate. Importantly, this conformational rearrangement of the 1A–2A inter-domain linker (W380 and R381) during nucleotide binding causes a significant ∼4 Å shift of the N-terminal end of the α9 helix, which in turn induces a structural rearrangement of the 2B domain.

Structural rearrangement of the 2B domain (described in detail below) induces changes at the inter-domain 2A–2B contact area, allowing the segment β18-η7-β19 comprising helicase motif V (see Supplementary Figure S1), to reach the γ-phosphate of ATP, represented in the ADP•AlF_4_^−^ structure by the AlF_4_^−^ moiety (Figure 2C). Accordingly, the ATP γ-phosphate and Mg^2+^ ion interact with G559 in motif V. At this point, the γ-phosphate attains its status as the focal point for the ensuing chemo-mechanical reaction linked to DNA translocation and unwinding, relaying conformational rearrangements to the whole protein and the 2B domain in particular.

Comparing the 1.13 Å hPIF1-AMP-PNP structure (Figure 2D, left) with the 2.0 Å ScPif1 ADP•AlF_4_^−^ structure (Figure 2D, centre), suggests that the water molecule hydrogen bonded to E307 of the Walker B motif (motif II) and arrowed in Figure 2D, is likely the ‘attacking water’, that makes a nucleophilic attack on the γ-phosphate to initiate ATP hydrolysis in the conventional model for ATP hydrolysis (see for example Supplementary Figure S1A in (46)). In the conventional model, E307 would act as a base removing a proton from the ‘attacking water’ giving a more associative model for ATP-hydrolysis initiated by converting the ‘attacking water’ to an ‘attacking OH^−^ ion’. However, a comparison of our 1.13 Å AMP-PNP structure with our 1.44 Å apo structure (Supplementary Figure S2) shows that the binding of the nucleotide induces movement of the Walker A motif (residues ^228^GSAGTGKS^235^). It is also possible that movement of the Walker A motif could cause the main-chain N–H of G231 (Figure 2D, left) to directly protonate the bridging oxygen of ATP (46). Interestingly, the ATP-binding pocket in the 1.13Å human PIF1-AMP-PNP structure more closely resembles that in the yeast Pif1-ADP•ALF_4_^−^-ssDNA complex and much of the water structure is conserved (Figure 2D, right), while there are substantive movements when the human hPIF1 apo and AMP-PNP structures are compared (Supplementary Figure S2A–C). A fully dissociative ‘wellington-boot’ type model (47) for ATP hydrolysis by the Walker A motif is tentatively proposed in Supplementary Figure S2D–F. This proposed mechanism has some similarities to that previously proposed for an unrelated ATP*ase* (see Figure 5 in (46)).

### Protein conformational changes during ATP binding and hydrolysis

**Figure 3.**
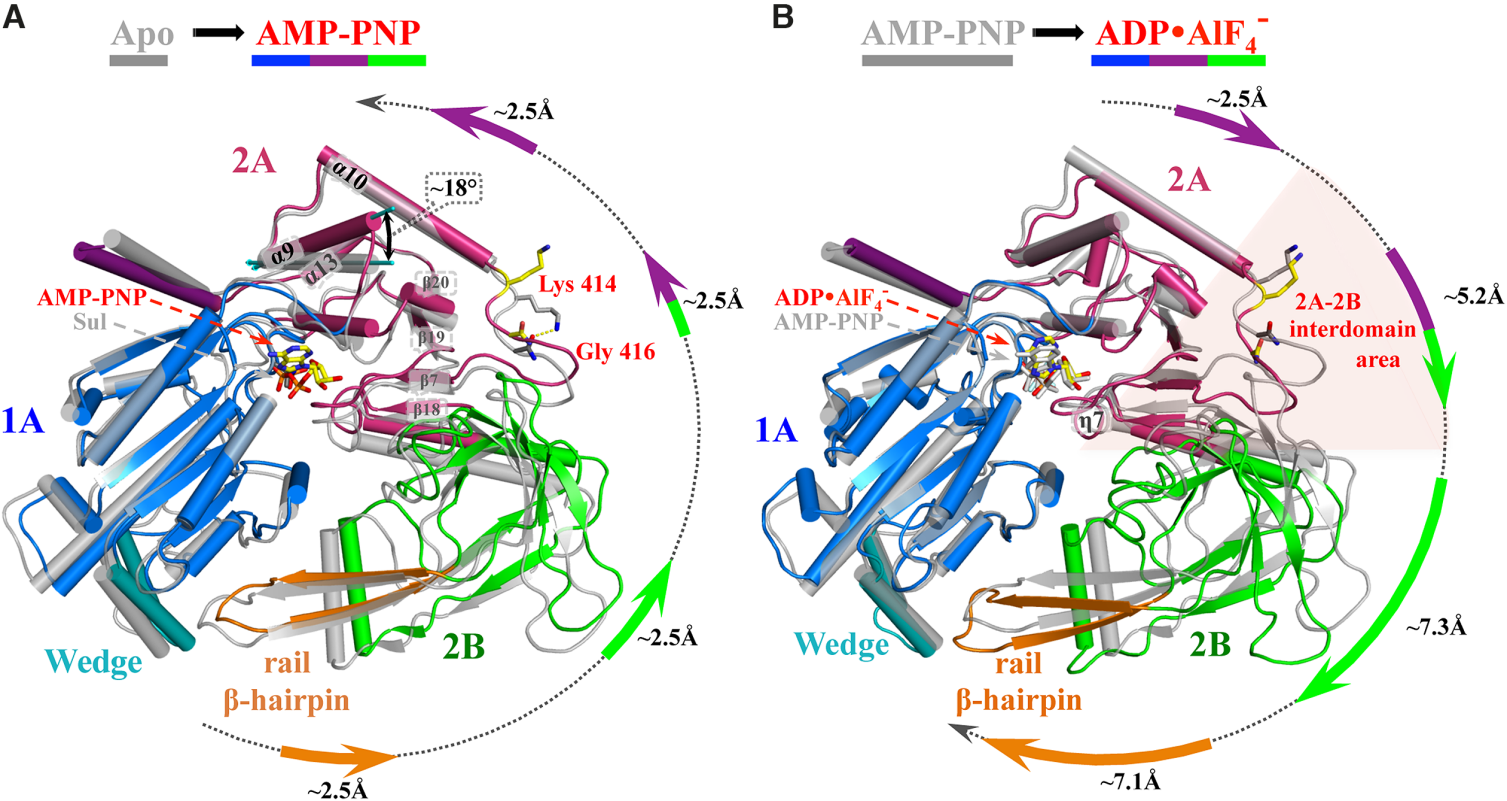
Structural rearrangements during ATP binding and hydrolysis. Structures shown in cartoon representation were superposed via the 1A domains. Ligand positions and secondary structural elements α9, α10, α13 and η7 and the β-sheet β20–β19–β7–β18 are indicated. K414 and G416 are represented as stick to illustrate their interaction. The direction and magnitude of the positional shifts in the domains, measured at the periphery of the structures, are indicated by the arrows in domain colours. (**A**) Changes upon nucleotide triphosphate binding represented by the AMP-PNP ligand. Apo-hPIF1 is shown in grey and the hPIF1-AMP-PNP structure with domains coloured as in Figure 1. The ∼18° angular displacement of α9, corresponding to conserved motif A of the RecD helicase subfamily, is indicated. (**B**) Structural transitions during ATP hydrolysis modelled with the hPIF1-AMP-PNP structure in grey and the ADP•AlF_4_^−^ bound structure in domain colours. The 2A–2B interdomain area is depicted by the pink shading.

ATP binding results in a 4 Å movement of the N-terminus of helix α9 (helix α9 corresponds to the conserved motif A, Supplementary Figure S1) while the C-terminus remains relatively fixed to α13, which is immobilized by interactions (R584) with the bound nucleotide. The immobilization of the α9 C-terminus is ensured by H-bonds between conserved residues R395 (motif A) and D343 (motif III). The corresponding angular displacement is ∼18° (Figure 3A). The N-terminus of α9 pushes the α10 helix aside, stretching the adjacent segment (residues 412–417, domain 2A) and breaking the hydrogen bond between the side chain of K414 and the main chain carboxyl of G416 (Supplementary Figure S3). Consequently, loop 412–417 becomes more flexible and allows a distortion of the β-sheet β20–β19–β7–β18 and adjustment of its interactions with the hydrophobic core of domain 2A. Comparing the AMP-PNP structure to apo-hPIF1, the observed overall structural changes during ATP binding are small, corresponding to a ∼2.5 Å anti-clockwise shift of the 2A and 2B domains, as depicted in Figure 3A. Notably, despite sequence conservation in the inter-domain area the level of sequence similarity does not allow identification of conserved interactions responsible for the structural rearrangements. As such, it is likely that conformational changes are conditioned by integral characteristics of the structure, such as flexibility and formation of hydrophobic clusters.

The increased flexibility of the 2A–2B interdomain area, shaded pink in Figure 3B, prepares the active site for nucleotide hydrolysis. In the post hydrolysis state, represented by the ADP•AlF_4_^−^ structure, G559 of motif V interacts with the γ-phosphate of ATP as described above. Changes in the ATP binding site, focused on interactions with the γ-phosphate, are relayed to significant clockwise movements at the periphery of the structure, as depicted in Figure 3B. This breaks a set of H-bonds which controls the relative orientations of the 2A and 2B domains, allowing the rotation of the 2B domain necessary for formation of the ssDNA binding cleft (26,27), including an H-bond between the conserved R515 and the carboxyl group of D418 (Supplementary Figure S3). Significantly, there is a ∼7.1 Å displacement of the rail β-hairpin (orange in Figure 3B) towards the wedge domain. Supplementary Movie S1, based on the solved apo-, AMP-PNP and ADP•AlF_4_^−^ bound hPIF1 structures shows the structural rearrangements during ATP binding and hydrolysis.

### A model of hPIF1 in complex with DNA

**Figure 4.**
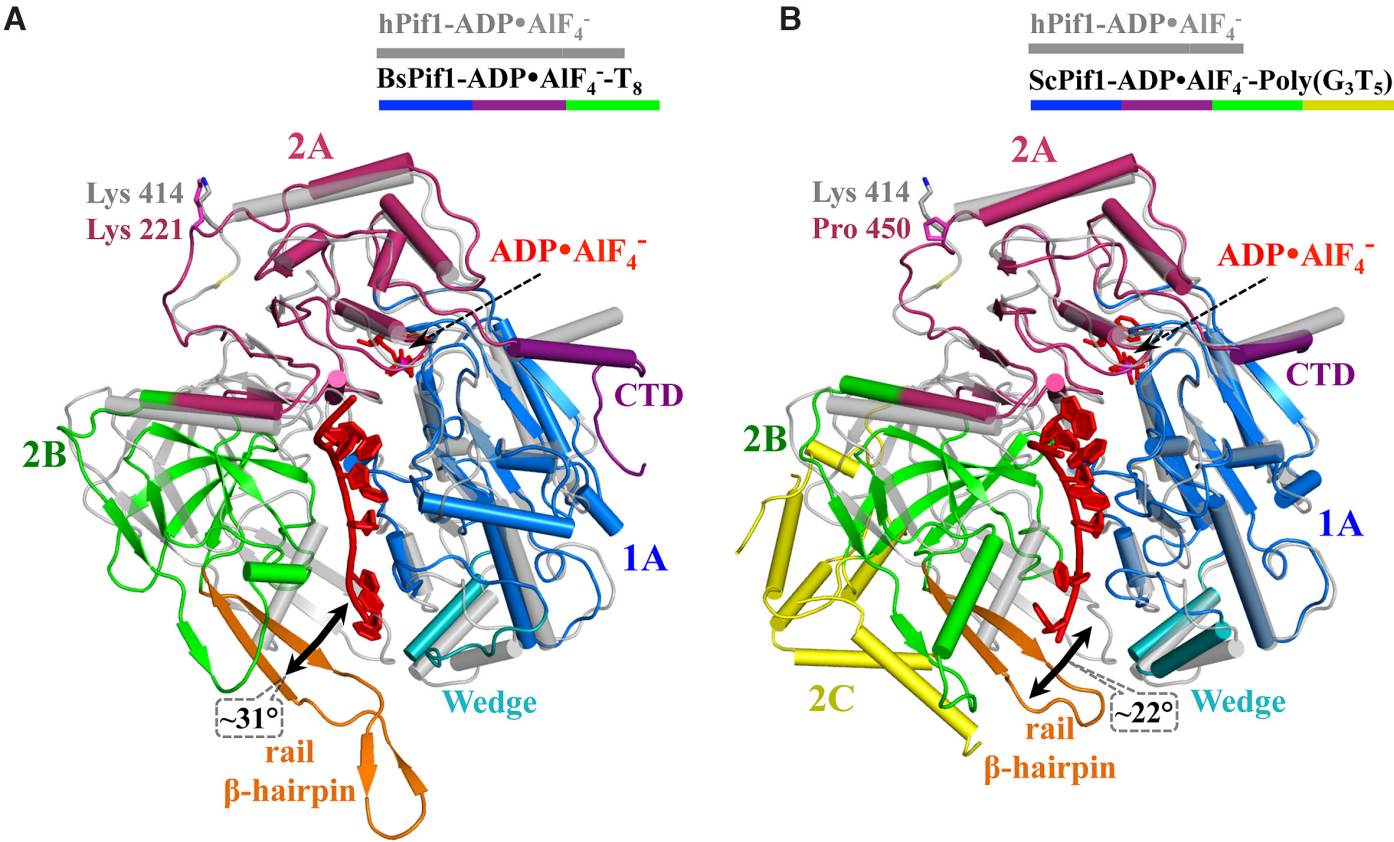
A comparison of the hPIF1-ADP•AlF_4_^−^ structure with structures of yeast and *Bacteroides* Pif1 bound to ADP•AlF_4_^−^ and ssDNA. The hPIF1-ADP•AlF_4_^−^ structure is shown in grey and the BsPif1 (**A**) and ScPif1 (**B**) nucleotide and ssDNA bound structures with the corresponding domains coloured (as for hPIF1, Figure 1). Structures are orientated to depict the ssDNA binding channel in side view with the ssDNA shown in red. hPIF1 K414 and the corresponding residues in Bs (K221) and ScPif1 (P450) are represented as stick models. The large relative movement and angular displacement of domain 2B (green) induced by simultaneous binding of ADP•AlF_4_^−^ and ssDNA is indicated in each case. Note the absence of a helix corresponding to hPIF1 α5 in the BsPif1 wedge domain and the insertion of the large domain 2C (yellow) in ScPif1.

**Figure 5.**
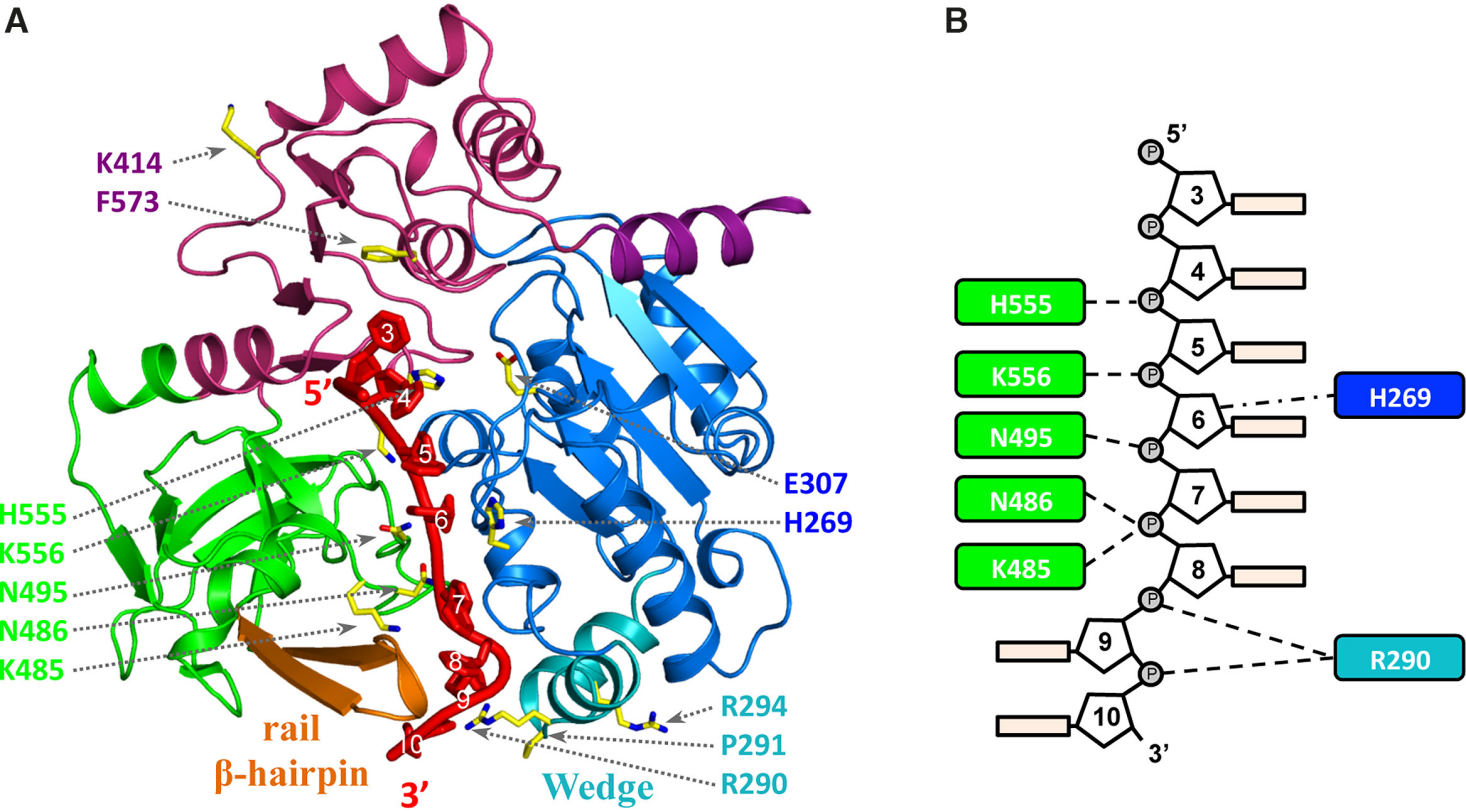
The hPIF1 DNA model with amino acid substitutions mapped. (**A**) The hPIF1-ssDNA structure showing the modelled bound ssDNA in red. The residues selected for substitution (Table 1) are represented as sticks model and numbered in domain colors. (**B**) A schematic of the proposed interactions with ssDNA, based on analysis of the hPIF1-ssDNA model, of the residues selected for substitution. The numbering of the bases is based on those observed in the ADP•AlF_4_^−^ and ssDNA bound BsPif1 structure (5FHD).

The hPIF1 structures and the yeast and *Bacteroides* spp. ssDNA-bound Pif1 structures (26,27,35) are in different conformations, with a large relative movement of domain 2B (Figure 4A and B). When the ADP•AlF_4_^−^ and ssDNA-bound and DNA-free conformations of BsPif1 structures are compared (26,27), it is seen that the conformational change involves a complicated rearrangement of residues in the 2A–2B inter-domain area, including a movement of the N-terminus of the α11-helix relative to the SH3 domain. Our attempts to co-crystallize hPIF1 with ssDNA, using accurately determined ratios of protein (see materials and methods, protein expression and purification) to DNA did not result in diffracting crystals, as has been the case previously (26,27). In the absence of structural data for a hPIF1–ssDNA complex, we constructed a model. An initial model was made by superposing domains from the 1.13 Å human complex with AMP-PNP onto the equivalent domains in the 2.0 Å *Bacteroides* Pif1 complex with ssDNA and ADP•AlF_4_^−^ bound (Supplementary Figure S4). A second hPIF1HD-ssDNA model (Figure 5A) was made when coordinates for the more closely related ScPif1 (sequence identity 44% for helicase core) in complex with ssDNA and ADP•AlF_4_^−^ (35) became available (Supplementary Figure S4). The two models are essentially the same, and henceforth referred to as ‘the model’, although there is a small shift in the DNA position and in the position of domains 2B (the SH3 domain). The model also confirms that the overall configuration and position of critical conserved amino acids (Figure 5B) in the ssDNA binding channel are likely to be conserved in Pif1 family helicases.

### Mutational analysis of DNA binding

The hPIF1-ssDNA model (Figure 5A) along with the primary sequence alignment of Pif1 proteins (Supplementary Figure S1) identifies specific residues that may have roles in engaging ssDNA. To test the model, we selected residues (Figure 5 and Table 1) for functional analysis in DNA-binding and unwinding assays. Three residues from the ‘separation wedge’ (domain 1B) at the entrance to the ssDNA binding channel and conserved residues therein were mutated. As noted above, the structure and sequence of the Pif1 wedge region is variable. In BsPif1, ssDNA has been shown to bend sharply near the wedge domain as it enters the ssDNA binding channel, while basic residues in the Lys-Arg rich wedge of ScPif1 have been implicated in G4 DNA binding (35). We therefore mutated the two arginine residues in the hPif1 wedge, R290 and R294, as well as P291 separating helices α5 and α6 for testing in functional assays.

**Table 1.** Residues mutated in hPIF1HD for biochemical analysis of purified proteins. The region or functional site within which the substitutions were made is indicated with reference to observations made on BsPif1 (26,27). Corresponding residues in ScPif1 and BsPif1, based on primary sequence alignments (Supplementary Figure S1), are given. Note the lack of sequence conservation in the separation wedge

	hPIF1 mutation	Domain	Motif/functional site	BsPif1 residue	ScPif1 residue
1	R290A	1B	Separation wedge	S89	S325
2	P291A	1B	Separation wedge	E90	R326
3	R294A	1B	Separation wedge	V93	L329
4	H269A	1A	ssDNA binding channel	H68	H393
5	E307Q	2B	Walker B, ATP hydrolysis	E106	E342
6	N495A	2B	ssDNA binding, C motif	N296	N533
7	H555A	2A	ssDNA binding channel	H361	H705
8	K556A	2A	ssDNA binding channel	K362	K706
9	F573A	2A	ssDNA binding channel	F379	F723
10	K485E	2B	B motif	V287	K525
11	N486A	2B	ssDNA binding, B motif	N288	N526
12	K414A	2A	SO_4_ binding (apo hPIF1 structure)	K221	P450

We also mutated K485, N486 and N495 of the poorly characterised B (residues 477–486) and C (residues 494–499) motifs within domain 2B (Supplementary Figure S1) that are characteristic of the SF1B RecD helicases sub-family (2,23). K485, N486 and N495 are in exposed loops where a sulphate ion is located in the apo-hPIF1 structure (SUL_2_ in Figure 1B). In bacterial Pif1, the large movements of domain 2B in response to substrate binding position residues of the B and C motifs in the ssDNA binding channel (26,27), as in our hPIF1-ssDNA model. We therefore reasoned that a decrease in ssDNA binding and catalytic activity of such mutants would be consistent with a similar movement and functional activity of these motifs in hPIF1. Residue K414 was also selected for analysis and we also included the E307Q Walker B motif variant as a control. K414 is also in the vicinity of a bound sulphate (SUL_3_ in Figure 1B) and is conserved between mammalian and bacterial Pif1, but it is not in the ssDNA binding channel revealed in Bs and ScPif1 structures (26,27,35). Based on our analysis described above, the alanine substitution would preclude formation of a hydrogen bond and leave the linker region (residues 412–417) more flexible. All variant proteins were expressed and purified as described for the wild-type hPIF1HD protein construct, hereafter referred to as wild-type hPIF1.

**Figure 6.**
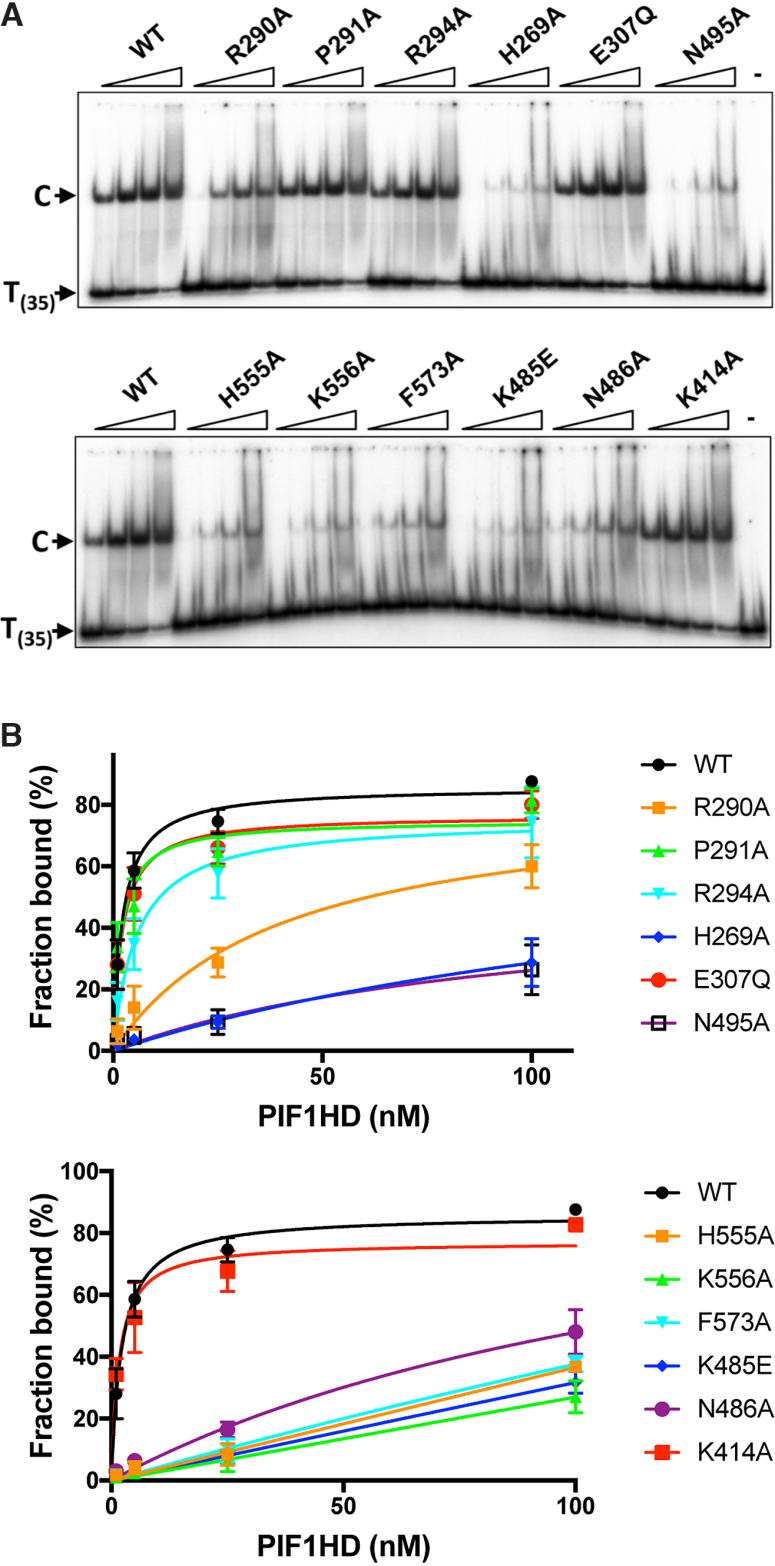
Binding of hPIF1 and variant proteins to oligo T_(35)_. (**A**) Products of ssDNA binding reactions (0.4 nM radiolabelled T_(35)_ substrate, 1, 5, 25 and 100 nM hPIF1) were resolved by poly-acrylamide gel electrophoresis. The primary protein–DNA complex ‘C’ is indicated as is the mobility of free DNA. The final lane in each panel shows the mobility of free substrate (no hPIF1 protein). (**B**) Binding data were quantified following phosphorimaging. Graphs of fraction bound plotted against protein concentration analysed by nonlinear regression using a single binding site model, *n* = 4 experimental repeats, mean and standard deviation delimited by the error bars shown.

Previously, we have observed that in the absence of nucleotide cofactors hPIF1 forms a single discrete ssDNA complex in gel-shift assays (EMSA) with polydeoxynucleotide substrates greater than 30 bases (29). Here, ssDNA binding reactions were assembled at pH 7.2 and 135 mM NaCl and binding of all proteins to a radiolabelled poly T_(35)_ substrate was assayed, Figure 6A. To quantify the bound fraction, we included all the shifted species distinguished by comparison with the control lane with substrate alone. With the wild-type protein, ∼85% binding was achieved at the highest protein concentration tested (100 nM) and an apparent *K*_d_ of 2.3 ± 0.3 nM was determined from the quantified data shown in Figure 6B using GraphPad PRISM. In the separation wedge, variants P291A bound T_(35)_ ssDNA with similar affinity to wild-type (1.9 ± 0.6 nM), while the binding affinity of R294 was reduced ∼2–3-fold (apparent *K*_d_ 5.7 ± 1.6). Binding of variant R290A was reduced ∼10–20-fold (apparent *K*_d_ 40.4 ± 14 nM), although the failure to reach near saturation binding precludes the determination of a more accurate apparent *K*_d_ value. Variants E307Q (Walker B motif) and K414A also bound ssDNA at close to wild-type levels (apparent *K*_d_ values of 3.6 ± 0.5 and 1.6 ± 0.4 nM respectively). All amino acid substitutions in the ssDNA binding channel including F573 and those from the B and C motifs (residues K485, N486 and N495A) result in large decreases in ssDNA binding affinity, with variant K556A showing the greatest defect. As above, insufficient binding extents (Figure 6B) preclude the determination of accurate comparative apparent *K*_d_ values, but the data indicate at least a 50-fold decrease in ssDNA binding affinity for all these variants.

### Helicase and ATP*ase* activity

**Figure 7.**
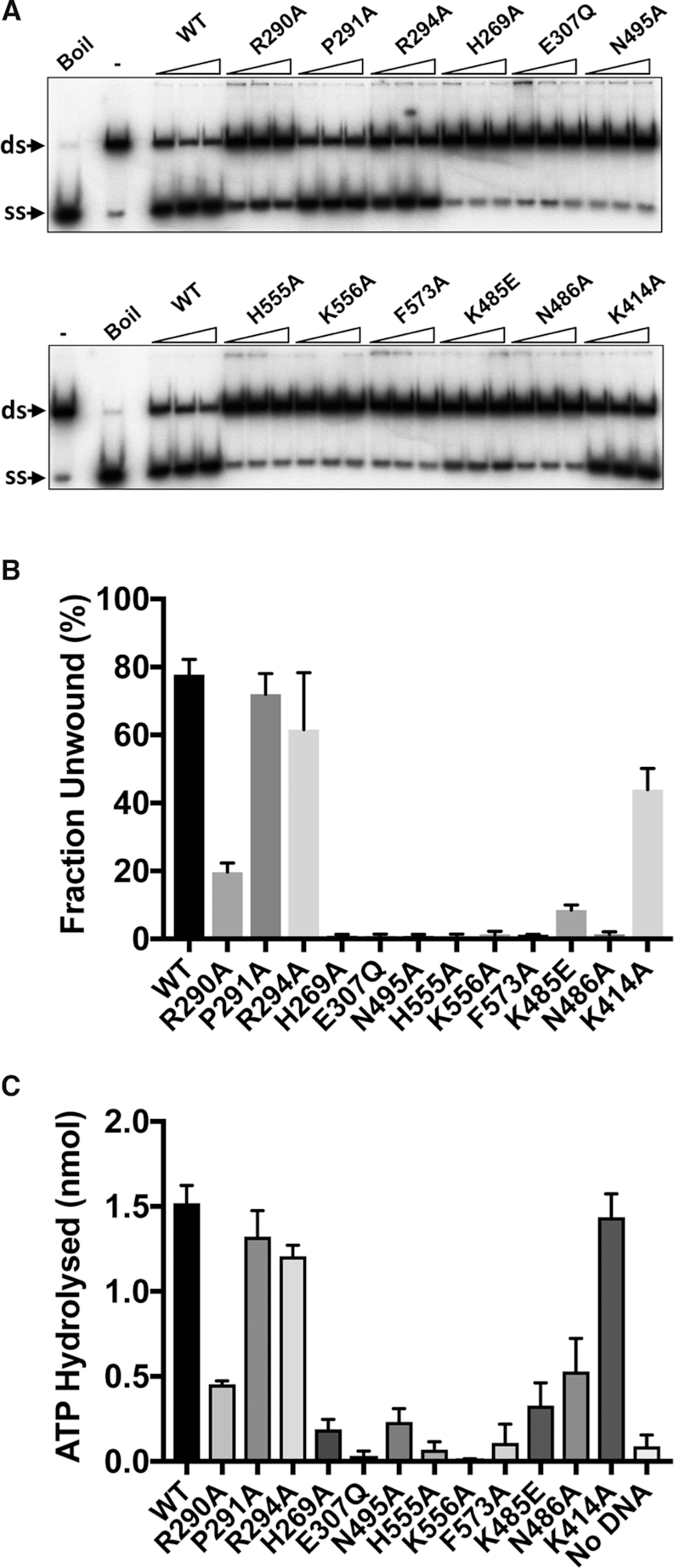
Enzymatic activities of hPIF1 and variant protein forms. (**A**) Displacement of a ^32^P radiolabelled 20 base ssDNA strand (ss) from a partially single- and double-stranded test substrate (ds). Reaction products (0.1 nM substrate, 3.75, 7.5 and 15 nM hPIF1) were resolved by poly-acrylamide gel electrophoresis for quantification following phosphorimaging. On the left of each panel the mobility of native substrate (ds, reaction with no hPIF1 protein) and the labelled ssDNA product generated by heat denaturation (Boil/(ss) product) are indicated. (**B**) Strand displacement activity (fraction unwound) for reactions with 7.5 nM hPIF1. *n* = 4 experimental repeats, mean and standard deviation delimited by the error bars are shown. A quantitative analysis of all titration points is shown in Supplementary Figure S5. (**C**) DNA dependent ATP*ase* was determined at 100 nM hPIF1/200 nM T_(30)_ ssDNA after 10 min incubation (∼ 3% of substrate hydrolyse for wild type PIF1). *n* = 3 experimental repeats, mean and standard deviation delimited by the error bars are shown

Wild-type and variant hPIF1 proteins were tested in an ATP-dependent helicase assays for their ability to displace a 20 base radiolabelled oligonucleotide from a partially single- and double-stranded test substrate. Unwinding reactions were performed at low salt concentration (20 mM NaCl) as the strand displacement activity of hPIF1 is significantly inhibited at higher salt concentrations and the protein inactive in unwinding assays performed at ∼50 mM NaCl. The inclusion of 0.1% NP40 was also required for optimal activity. Reaction products were separated by polyacrylamide gel electrophoresis and quantified by phosphorimaging. The protein concentration range chosen was at the upper-end of the range where maximum unwinding is observed for wild-type protein (Figure 7A and Supplementary Figure S5) and where further increases in protein concentration result in decreased unwinding efficiency. The quantified data for the intermediate titration point is shown in Figure 7B, while Supplementary Figure S5 shows the analysis for all titration points. The magnitude of the defects observed in the helicase activity (Figure 7A and B) of hPIF1 variants R290A (25% of wild-type), P291A and R294A (>80% of wild-type) reflect the defects observed in ssDNA binding. As expected, variant E307Q (Walker B motif, Figure 2D) was inactive in helicase assays. All amino acid substitutions in the putative ssDNA binding channel, including K485E, N486A (B motif) and N495 (C motif) resulted in complete or near complete abolition of unwinding activity. Variant K414 retained unwinding activity at ∼60% of the wild-type level.

hPIF1 is a ssDNA dependent ATP*as*e (29). We asked whether the observed defects in ssDNA binding would also result in reduced ATP*ase* activity. Like hPIF1 unwinding activity, ATP hydrolysis is inhibited with increasing salt concentration. In order to measure suitable levels of ATP hydrolysis, it is necessary to perform reactions at higher protein concentration relative to helicase assays (100 nM), with excess T_(35)_ ssDNA and at a reduced salt concentration (75 mM NaCl) relative to direct ssDNA binding assays (Figure 6). ATP hydrolysis was assayed for wild-type and variant forms. As expected hPIF1 E307Q, which is directly involved in ATP hydrolysis, had minimal measurable ATP*ase* activity. Variants P291A and R294 hydrolysed ATP at close to wild type-levels, while the extent of ATP hydrolysis by R290A was reduced ∼3-fold. Therefore, for the separation wedge region, the defects in ATP*ase* activity reflect the defect observed in ssDNA binding and helicase activity described above. All amino acid substitutions in the ssDNA binding channel resulted in variant hPIF1 forms with significantly impaired ATP hydrolysis. Again, there was a strong correlation between loss of ATP*ase* activity and the magnitude of the reduction in apparent ssDNA binding affinity, where variant K556A showed little or no ATP*ase* activity and N486A retained only ∼30% activity compared to wild-type. Variant K414 had wild-type ATP*ase* activity, as was the ssDNA binding activity observed in the assays described above (Figure 6).

### ssDNA strand annealing activity

**Figure 8.**
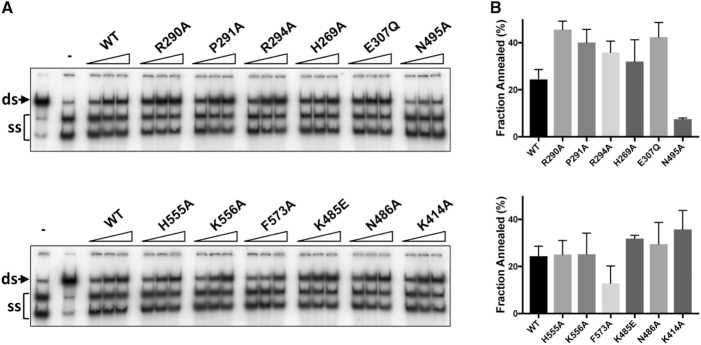
Strand annealing catalysed by hPIF1 and variant proteins. Reactions (50, 100, 150 nM PIF1) employed two oligonucleotides with 20 bases of complementary DNA (0.1 nM, both strands labelled). The electrophoretic mobility of the partially single- and double-stranded product (native/ds) and the heat denatured substrate (–, no hPIF1 reaction) are indicate in the lanes to the left of each gel image. Quantified data (150 nM hPIF1), *n* = 4 experimental repeats, mean and standard deviation delimited by the error bars, are shown to the right of each panel. A quantitative analysis of all titration points is shown in Supplementary Figure S6.

The hPIF1 helicase core catalyses the annealing of complementary ssDNA strands in the absence of ATP/Mg^2+^ (29), but the mechanism of ssDNA strand annealing is unknown. We asked if the residues required for ssDNA binding and unwinding activity are also required for DNA annealing. Like the DNA unwinding activity of hPIF1, the DNA strand annealing activity is highly salt sensitive although, unlike helicase activity, not completely inhibited at high salt concentrations. Here, for direct comparison with the ssDNA binding assays, we performed DNA strand annealing assays at pH 7.2 and 135 mM NaCl and at higher protein concentration relative to helicase assays. To assess the ssDNA strand annealing activity of wild-type and variant hPIF1 proteins we purified a fork-like duplex substrate (20 base pairs) with ssDNA tails (5′ T55 and 3′ C30) and both strands end-labelled with ^32^P. The duplex was heat denatured prior to addition to the annealing reaction and reaction products were separated by polyacrylamide gel electrophoresis for visualization and quantification. Wild-type and mutant proteins showed increasing strand annealing activity with increasing protein concentration over the range tested, Figure 8A. Figure 8B shows the quantified data for the intermediate protein concentration, while Supplementary Figure S6 shows the quantified data for all titration points. With the exception of mutants N495A (C motif) and F573A all mutants catalysed strand annealing at or significantly above wild-type activity levels across the range of protein concentrations tested.

### Binding of tetramolecular G4 DNA

**Figure 9.**
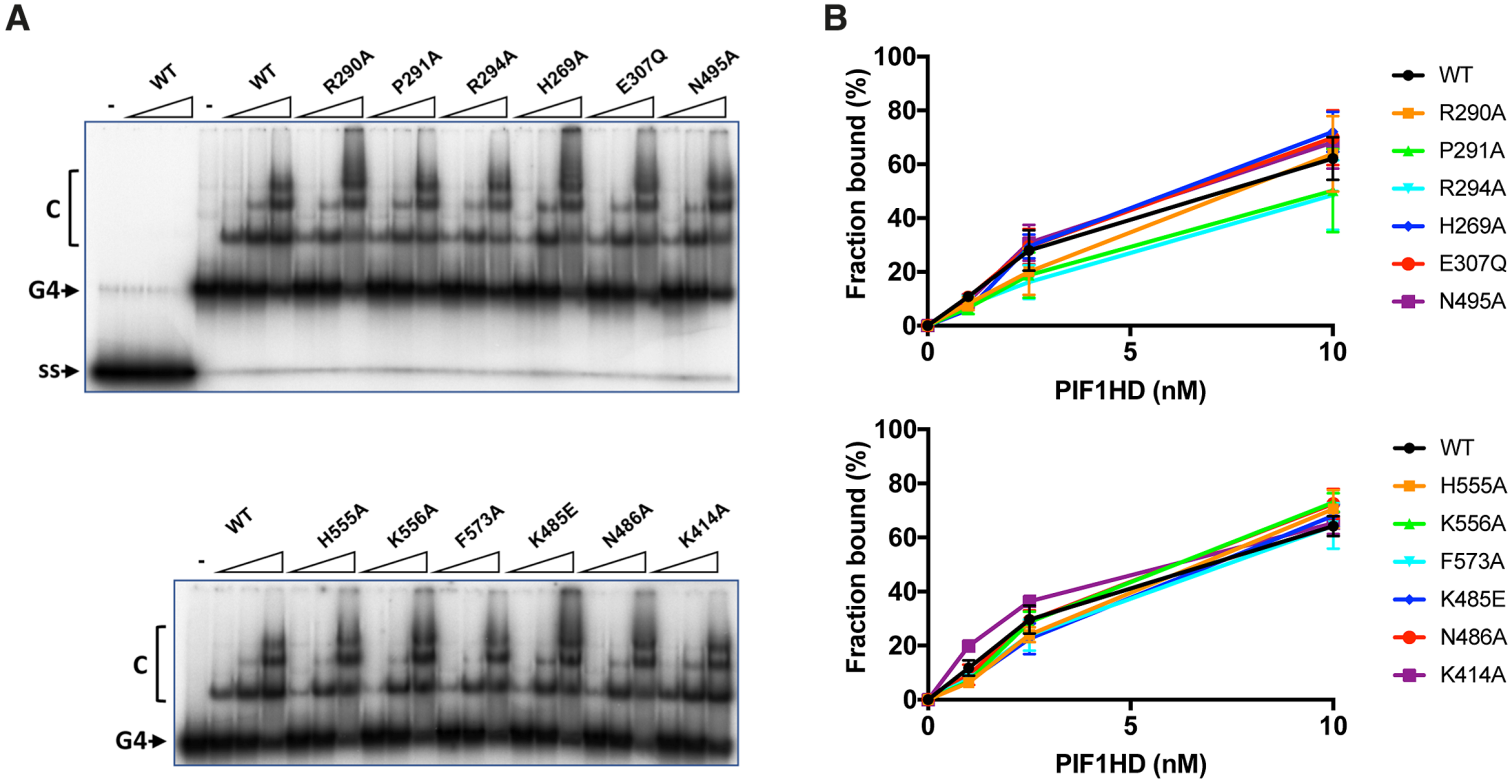
Binding of hPIF1 and variant proteins to tetramolecular G4 DNA. (**A**) Products of G4 DNA binding reactions (0.1 nM G4 DNA substrate; 1, 2.5, 10 nM hPIF1) were resolved by polyacrylamide gel electrophoresis and quantified following phosphorimaging. The first four lanes of the top panel demonstrate minimal binding of wild-type hPIF1 to the single-stranded precursor of the G4 substrate (ss). (**B**) Quantification of the binding data for *n* = 3 experimental repeats, mean and standard deviation delimited by the error bars.

A G4 DNA binding activity of hPIF1 resides in helicase core (29) and this activity can be readily visualized by EMSA. However, the structural determinants of G4 DNA binding have not been identified. Here we asked whether the same residues that impact on ssDNA binding and dsDNA unwinding activity are also necessary for G4 DNA binding. In the absence of nucleotide cofactors hPIF1 demonstrates a substantially higher affinity for a synthetic tetramolecular G4 DNA substrate with 5′ ssDNA tails compared to its single-stranded DNA precursor (ref. 29 and Figure 9A). Here, the reaction conditions used were identical to those used in ssDNA binding reactions (Figure 6), except that glycerol was omitted and replaced with 2% w/v PEG 8000, which was necessary to improve resolution of the bound products. The pattern of G4 DNA binding observed for wild-type shows predominantly a single complex at low protein concentrations and then the formation of higher-order species at higher protein concentrations, indicating a binding mode other than a simple bimolecular binding reaction, as observed for the ScPif1 protein (48,49). To quantify G4 DNA binding extents (Figure 9B), all complexes were included in the bound fraction, including the minor fraction of material retained in the wells at the highest protein concentrations.

Overall, the results of hPIF1 binding to G4 DNA were significantly different to the results observed for ssDNA binding (Figure 6). Only small reductions in binding extents (less than a 2-fold at the lowest protein concentration tested, 1 nM hPIF1) were observed, which reached statistical significance (*P* < 0.05) only for the variant H269A, H555A and K556A (Figure 9B). Curiously, variant K414A consistently displayed an increase in G4 DNA binding activity relative to wild type at the lower protein concentrations tested (*P* = 0.01 at 1 nM hPIF1).

## DISCUSSION

Our understanding of helicase mechanisms is incomplete since, for any one enzyme, structural information is only available for a limited number of conformational states. Here, we have obtained structures of hPIF1 representing three steps along the chemical reaction coordinate of ATP hydrolysis. Comparing these structures revealed the structural changes associated with an idle cycle of ATP hydrolysis and suggested how they could be involved in helicase action. First, roles of conserved residues in SF1 motifs III-VI, as well as the Walker A (motif I) and Walker B (motif II), are clearly defined in the chemo-mechanical chain of events. Second, events at the ATP binding site and in particular the ATP γ-phosphate are relayed to positional and conformational adjustments in defined protein segments. Third, such structural changes result in an increased flexibility of the protein structure that could affect protein–DNA interactions. The increased flexibility of the ADP•AlF_4_^−^ bound hPIF1 is clearly indicated in the overall atomic temperature factors of the refined models (Supplementary Table S1).

Although Pif1 proteins can bind ssDNA in the presence and absence of nucleotide cofactors, so far Pif1-ssDNA structures have only been obtained with ADP•AlF_4_^−^ bound. Indeed, the higher affinity of BsPif1 towards ssDNA observed with the ADP•AlF_4_^−^ transition-state mimic may have facilitated determination of the protein-DNA complex X-ray structures (26,27). ADP•AlF_4_^−^ also induces an increase in hPIF1 ssDNA binding affinity, while in the presence of AMP-PNP (ground-state mimic) or ADP (product) ssDNA binding affinities are lower than those observed in the absence of cofactor (Supplementary Figure S7). Rotation of domain 2B is required for ssDNA binding and this is permitted due to the increased flexibility and conformational adjustments observed for Pif1-ADP•AlF_4_^−^ structures. In hPIF1, breaking the hydrogen bond between R515 and the main chain of D418 is an important component of this induced flexibility, for example. A likely explanation therefore for the increased ssDNA binding affinity observed with ADP•AlF_4_^−^ is the facilitation of the movement of the ssDNA binding residues of the B and C motifs in domain 2B to become an integral part of the ssDNA binding channel. Importantly, a ∼7.1 Å displacement of the rail β-hairpin (orange in Figure 3B) towards the wedge domain could also be utilized to split the DNA duplex. Although not directly visualized here, it is also likely that accompanying structural changes in the ssDNA binding channel modulated ssDNA binding events associated with translocation, as has been observed in the 3′–5′ SF1A helicase PcrA (50). Thus, structural rearrangements linked to nucleotide hydrolysis may synchronise duplex splitting with directional movement on ssDNA (Supplementary Movie S1).

Our hPIF1 structures allowed the generation and testing of a model for hPIF1 ssDNA binding. Although the model does not allow a precise structural and mechanistic interpretation of the data, significant conclusions can be made. All substitutions of conserved residues in the putative hPIF1 ssDNA binding channel resulted in a substantial ∼50-fold decrease in ssDNA binding affinity in the absence of nucleotide cofactors. With the exception of K485E, which retained ∼10% activity, all ssDNA binding channel mutants were inactive in helicase assays and had decreased DNA-dependent ATP*as*e activity, mirroring the results of direct ssDNA binding assays. However, variants K485E, N486A and N495A in the conserved B and C motifs of domain 2B retained elevated levels of DNA-dependent ATP*ase* activity compared to others. In the 2B domain of BsPif1, only N288 (hPIF1 N486, B motif) and N296 (hPIF1 N495, C motif) interact with DNA (26,27), forming hydrogen bonds with the phosphate backbone, as do the equivalent residues in ScPif1 (35). The residue corresponding to hPIF1 K485 in BsPif1 is V287 and K525 in ScPif1, neither of which interact with ssDNA in the structures determined (26,27,35). Lysine at this position is largely conserved in mammalian Pif1 proteins and RecD from *E. coli* and *D. radiodurans*. Mutation and deletion of the short linker region between the BsPif1 B and C motifs (27) significantly impairs DNA unwinding but not ssDNA binding and ATP*ase* activity. The BsPif1 substitution N296A (C motif) retained ∼60% ssDNA binding activity while for the corresponding substitution in hPIF1 (N495A) binding affinity is reduced ∼50 fold. Together, the mutational data indicate that the mode of ssDNA binding is substantially conserved in Pif1 proteins. However, they also indicate that the residues in the B and C motifs may make a greater contribution to ssDNA binding in the mammalian compared to bacterial Pif1 proteins.

The separation wedge, domain 1B, is at the entrance to the ssDNA binding channel. As noted above, the sequence and structure of the wedge is variable. The apical residues of the helical turns where strand separation is assumed to occur are ^87^KFSEYK^92^ in BsPif1, ^289^QRPGVR^294^ in hPIF1 and ^323^RRSRKH^328^ in ScPif1. Although several residues of the BsPif1 segment interact with ssDNA, which in the crystal structure (pdb code 5FHD) is observed to bend sharply as it enters the ssDNA binding channel, alanine substitution of the residues results in only modest reductions in ssDNA binding and DNA unwinding activity (26). In hPIF1, while variants P291A and R294A have near wild-type ssDNA binding and unwinding activities, R290A results in a significant decrease in ssDNA binding affinity (∼10–20 fold), unwinding (∼25% of wild-type) and DNA-stimulated ATP hydrolysis (∼30% wild-type). Our model places R290 but not P291 or R294 in contact with ssDNA. These data suggest that wedge domains of Pif1 proteins may be optimised differently, and hence caution should be exercised in extrapolating directly from microbial to human PIF1.

The mechanism of DNA strand annealing is unknown. Unexpectedly, all variant proteins other than F573A and N495A had annealing activities equivalent to or greater than wild-type. N495 (C motif), is on the outer surface of the DNA-free hPIF1 structures, and based on our ssDNA-ADP•AlF_4_^−^ models moves ∼16Å to become an integral part of the ssDNA binding channel. F573 is at the exit of the ssDNA binding channel (Figure 5) and binds the terminal 5′ residue of ssDNA in the BsPif1 structure. Without nucleotide cofactors high affinity hPIF1-ssDNA binding is only observed with poly-T oligonucleotides >30 bases (29). ScPif1 can interact simultaneously with two ssDNA molecules (22), suggesting that at least two low affinity binding sites exist that could allow interactions with long oligonucleotides or facilitate DNA strand annealing. Other direct and indirect observations indicate that SF1, SF2 (51,52) and hexameric helicase (53) engage both the translocating (active) and displaced (passive) ssDNA strands along distinct binding paths. Whether residues N495 and F573 have direct roles in ssDNA annealing and why mutations in the active ssDNA binding channel can enhance annealing activity awaits further structural information. In particular, structures of hPIF1 with forked DNA substrates bound should indicate all DNA binding surfaces involved in annealing or unwinding as well as the role of the rail β hairpin in duplex splitting, proposed from our structural observations described above.

Although it is unknown how Pif1 proteins bind G4 DNA some parallels could be drawn from the structure of the SF2 helicase DHX36 (RHAU, G4R1) bound to a 3′ ssDNA tailed G4 DNA substrate (54), where the G4 DNA is engaged by an α-helical motif at the entrance to the ssDNA binding channel. Indirect observations indicate that in ScPif1 G4 DNA is clamped at the entrance to the ssDNA binding channel in ‘pliers’ of two sets of positively charged residues (35). However, one arm of the plier is in domain 2C and absent in hPIF1 (and BsPif1), while the second is in the positively charged wedge. Of the three wedge residues implicated in ScPif1 G4 DNA binding, only one (R326) is at a similar position in the hPIF1 structure, residue R290. Analysis of hPIF1 G4 DNA binding demonstrated only small defects in G4 DNA binding for mutants in the ssDNA binding channel and R290. As such the data do not identify a G4-specific DNA binding segment in hPIF1 and indicate that the mode of G4 DNA binding is not strictly conserved. The increased G4 DNA binding of mutant K414A relative to wild-type observed at low protein concentrations we believe is likely related to overall protein conformational flexibility, as discussed above.

In conclusion, the structural analysis indicates how ATP binding and hydrolysis events are coupled to DNA binding. The mutational data indicate that the ssDNA binding channel is substantially conserved in all Pif1 proteins. However, sequence and structural variability in functional motifs (e.g. the wedge region), the presence of additional domains (e.g. the 2C domain insertion in ScPif1) and the analysis of variant hPIF1 forms presented here indicate that caution is required in extrapolating directly from the analysis of microbial Pif1 proteins to human. Further studies of hPIF1, in particular structural studies on complexes with DNA substrates and helicase activity inhibitors, are required to fully understand its mechanism and to establish possible routes for therapeutic intervention.

## DATA AVAILABILITY

The coordinates and structure factors of the apo hPIF1, hPIF1-AMP-PNP, hPIF1-AMP-PNP(Br) and hPIF1-ADP•AlF_4_^−^ structures have been deposited with the Protein Data Bank under accession codes 6HPT, 6HPH and 6HPQ and 6HPU respectively.

## Supplementary Material

Supplementary Data
