# Supplementary material for: Structural and functional analysis of the nucleotide and DNA binding activities of the human PIF1 helicase
Source: Nucleic Acids Res. 2019 Jan 30;47(6):3208–22. doi: 10.1093/nar/gkz028 (PMC6451128; doi:10.1093/nar/gkz028)
Supplement: Supplementary Data [file gkz028_supplemental_files.zip › Revised_SupplementaryData_Deghani.pdf]

Supplementary Figure S1.

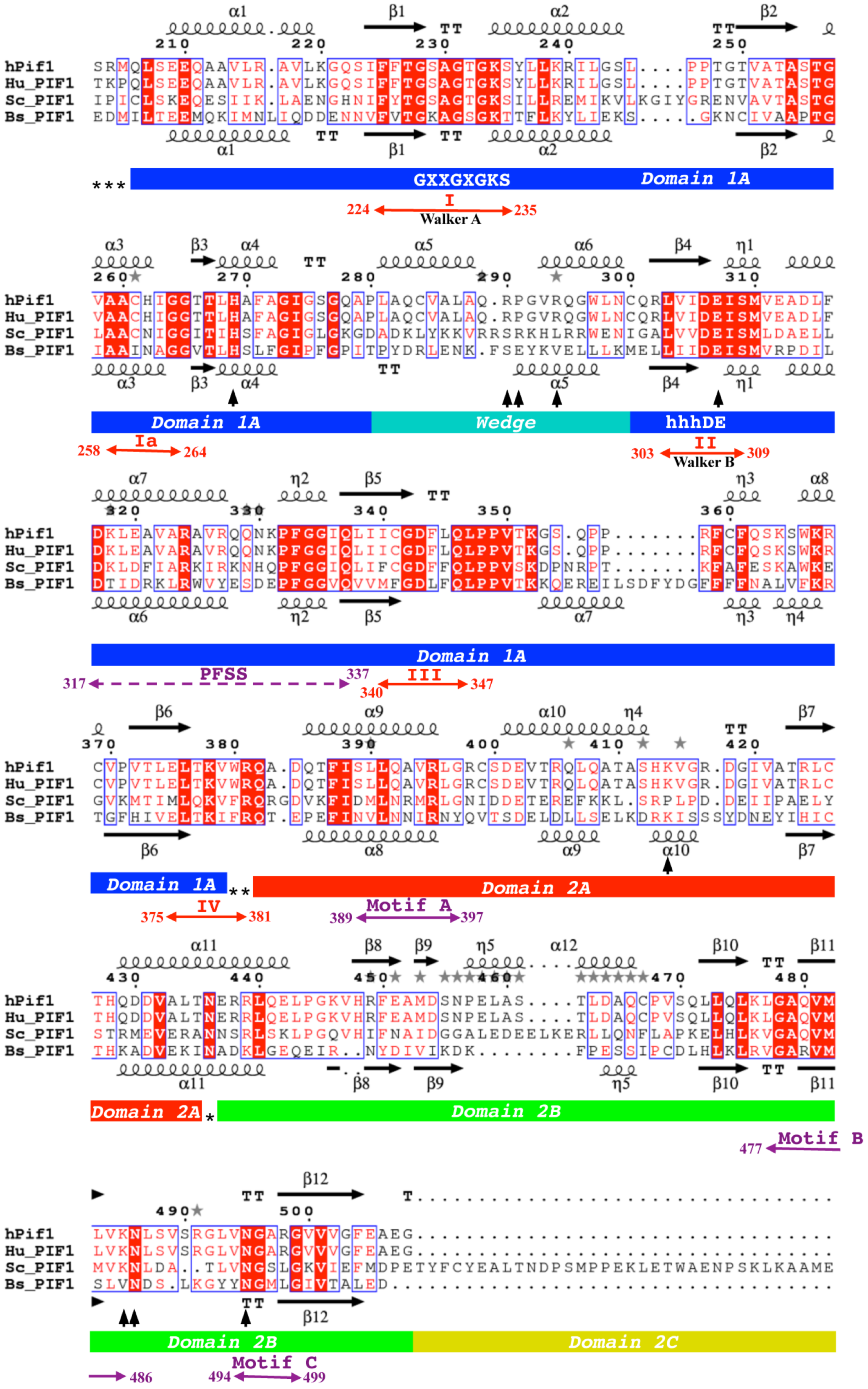

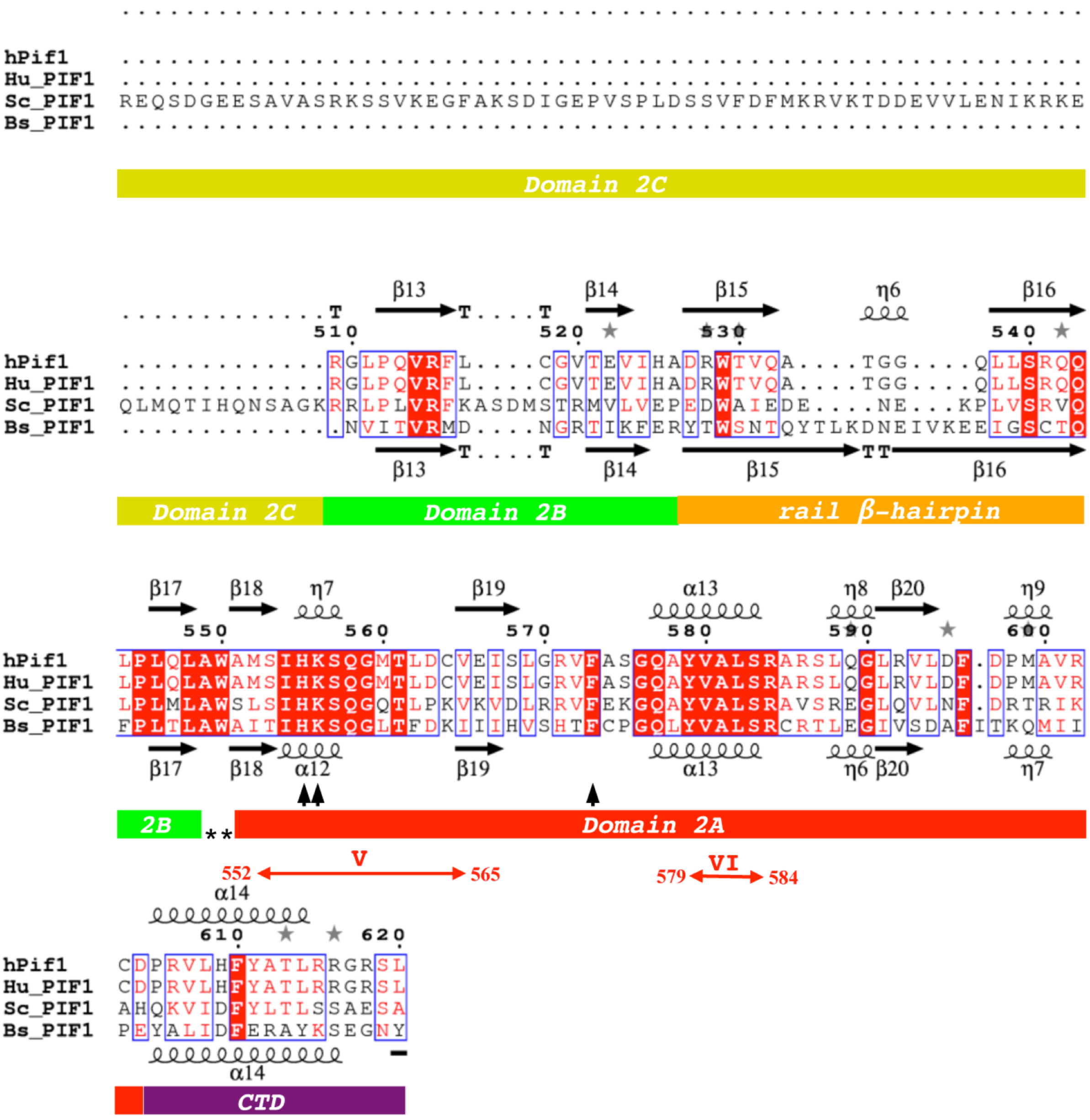

Figure S1. Pif1 sequence alignment. The first line, hPif1, is the sequence of the human Pif1 protein construct for which electron density was modelled in our structures. The human Pif1 constructs used start at residue 206 and are preceded by four residues from the tag (GSRM) but the electron density for the glycine is not visible. The sequence indicated as hPif1 is aligned against the native human sequence (Hu_PIF1) beginning at residue 203 (residues 203-205 aligning with SRM of the tag), as well as the corresponding residues for *Bacteroides spp*. (Bs_PIF1) and the yeast (Sc_PIF1) helicase domain. Identical residues are boxed in red, similar residues are red and non conserved residues are black. Regions framed in blue have a global similarity >0.7. Secondary structural elements are indicated as α1, β1, η1 (3/10 helix), TT (β turn) and dark grey stars are residue with alternative conformations. Note, linker regions between domains are indicated by *s in the domain definition line (below sequences). Walker A (GXXXXGK[TS]) and Walker B (hhhDE) sequence motifs are also shown in the domain line (h=hydrophobic residue). The twelve residues indicated by black arrows ↑ were mutated in this study (see Table 1, main manuscript). Top and bottom secondary structures are from ESPript 3.0 (Robert, X. and Gouet, P. (2014) Deciphering key features in protein structures with the new ENDscript server. *Nucleic Acids Res.* 42(W1), W320-W324. doi: 10.1093/nar/gku316) for the 1.13 Å AMP-PNP complex (hPIF1) and 5FTB (BsPif1), respectively. We define domain 1B (the separation wedge) as residues 280-300 (cyan in Fig. 1, main manuscript). The residue numbers above the alignment are from the native Hu_PIF1 sequence.

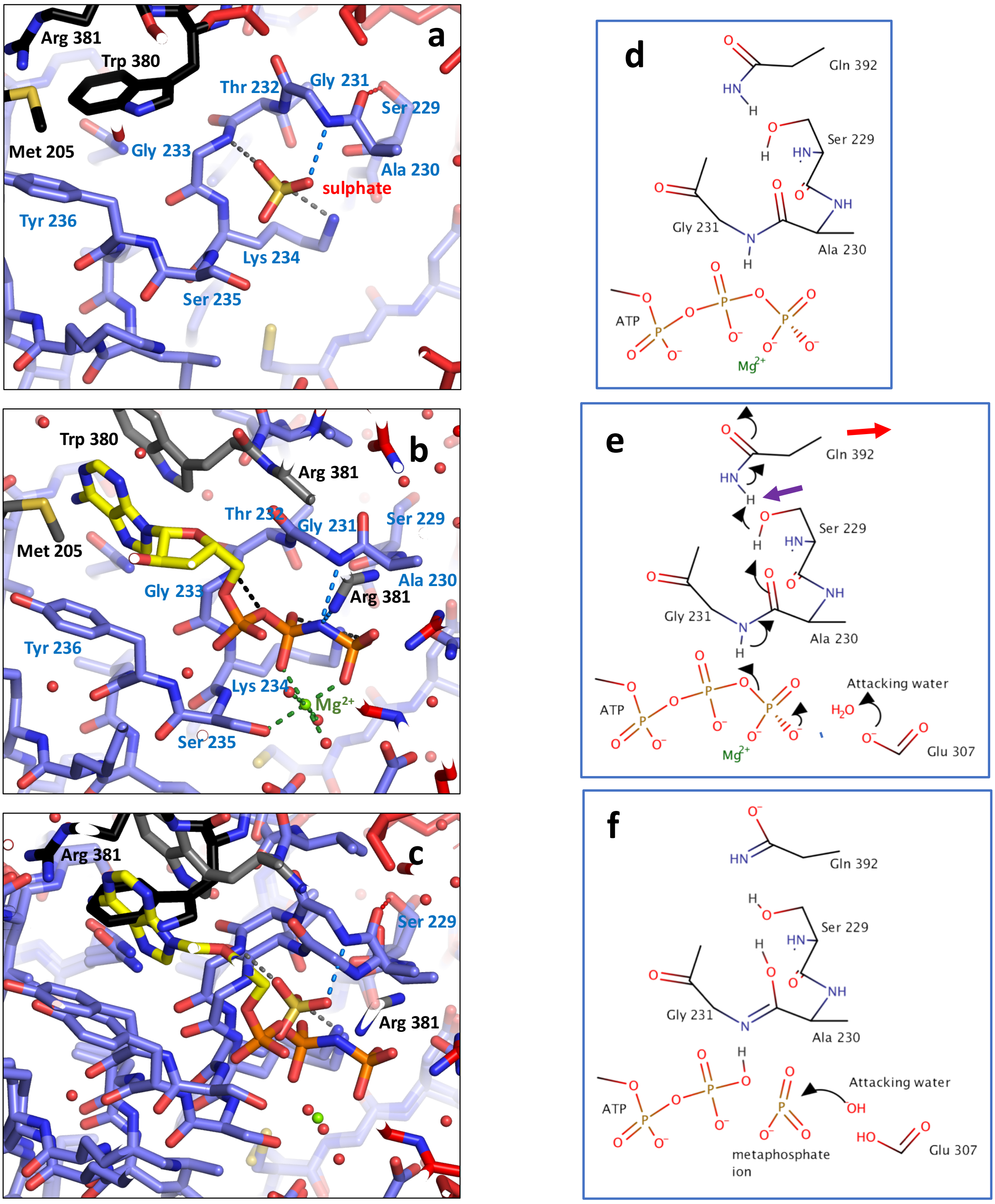

Supplementary Figure S2 – A comparison of the ATP binding site in the 1.44 Å apo and the 1.13 Å hPIF1-AMPPNP structures and a proposed fully dissociative 'Wellington boot remover' type ATP-hydrolysis mechanism. (a) In the 1.44Å apo structure a sulphate ion (SO_4^{2-}) interacts with N-Hs from G231, G233 and K234 from the Walker A motif (GSAGTGKS) in a similar manner to the β-phosphate in the AMP-PNP structure. Waters are not shown in **a**. Carbons are light blue in domain 1A and red in domain 2A, carbons are black in W380 and R381 (and in M205). **(b)** In the 1.13 Å AMP-PNP hPIF1 structure the nitrogen (blue atom) between the β- and γ-phosphates of AMP-PNP is some 3.0 Å from the main-chain NH of G231 from the Walker A motif (interaction shown by blue dotted line). For clarity only some interactions are shown, and the viewing slab has been narrowed so only the end of the side-chain of R381 and some of the main-chain is visible (see also Fig. 2D). Waters are shown as small red spheres, and the Mg^{2+} ion as a green sphere. **(c)** Structures in **a** and **b** are superposed based on domain 1 (blue carbons). **(d,e,f)** Interdomain movement causes Q392 to protonate S229 (from the Walker A motif) – leading to protonation of the bridging oxygen between the β- and γ-phosphates, and ATP hydrolysis via the formation of a discrete metaphosphate ion, which is then 'attacked' by the attacking water (OH^- ion - panel **f**). In panel **e** interdomain movement is about to cause Q392 to be moved about 2 Å to the right (red arrow), causing it to leave behind a hydrogen (purple arrow), rather as a Wellington boot is left behind when the foot bearing the Wellington boot is moved past a 'Wellington boot remover'. (Bax *et al.*, 2017, Agrawal *et al.*, 2013).

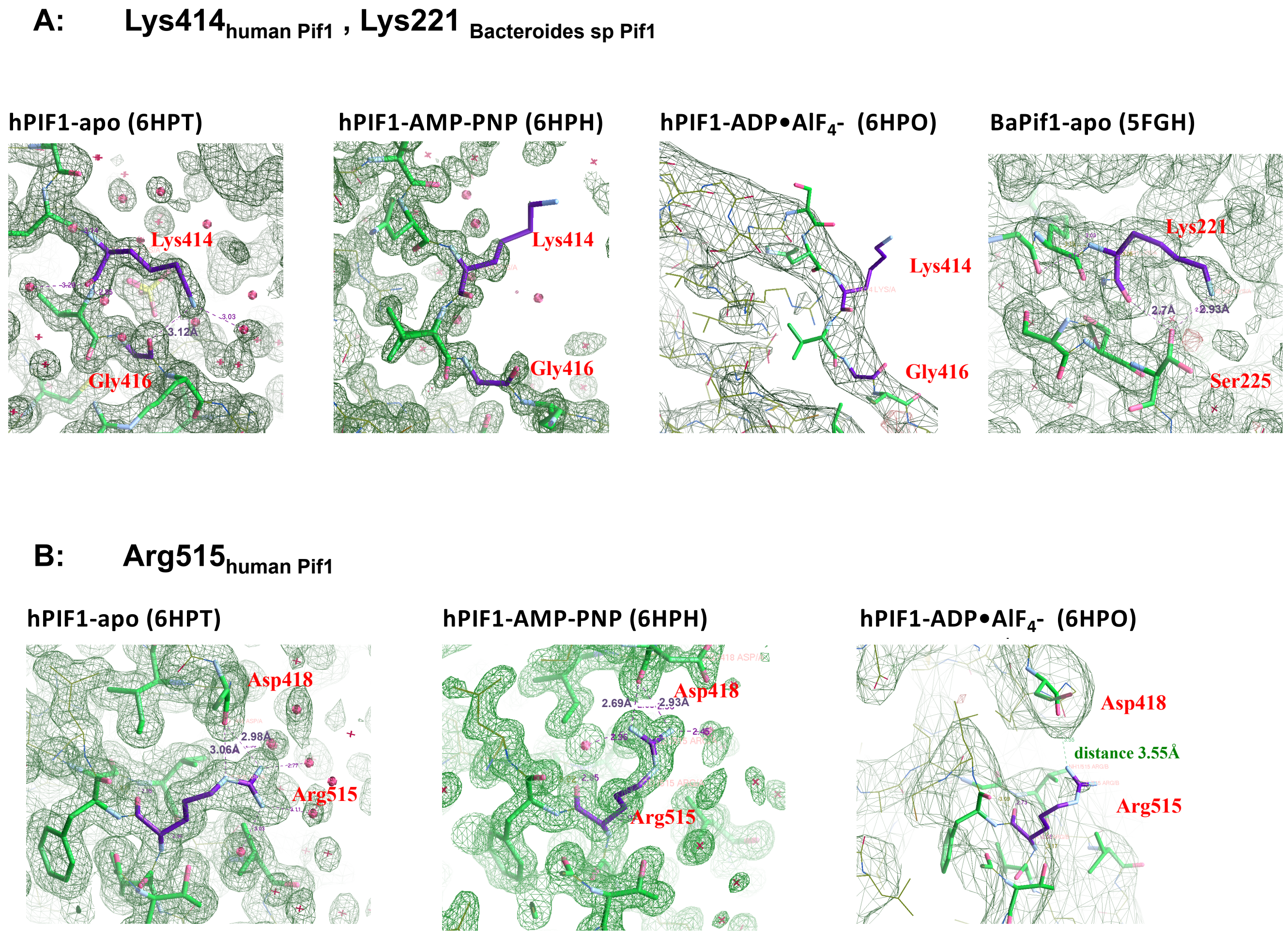

Supplementary Fig. S3. Conformational differences and hydrogen bond formation associated with different steps of ATP hydrolysis. Electron density maps, calculated with maximum likelihood weighted 2|Fo| - |Fc| coefficients, contoured at the 1σ level for **(A)** hPIF1 region at residues K414 and G416 and the corresponding residues in BsPif1 and **(B)** hPIF1 region at residues D418 and R515. Corresponding PDB codes are given in brackets.

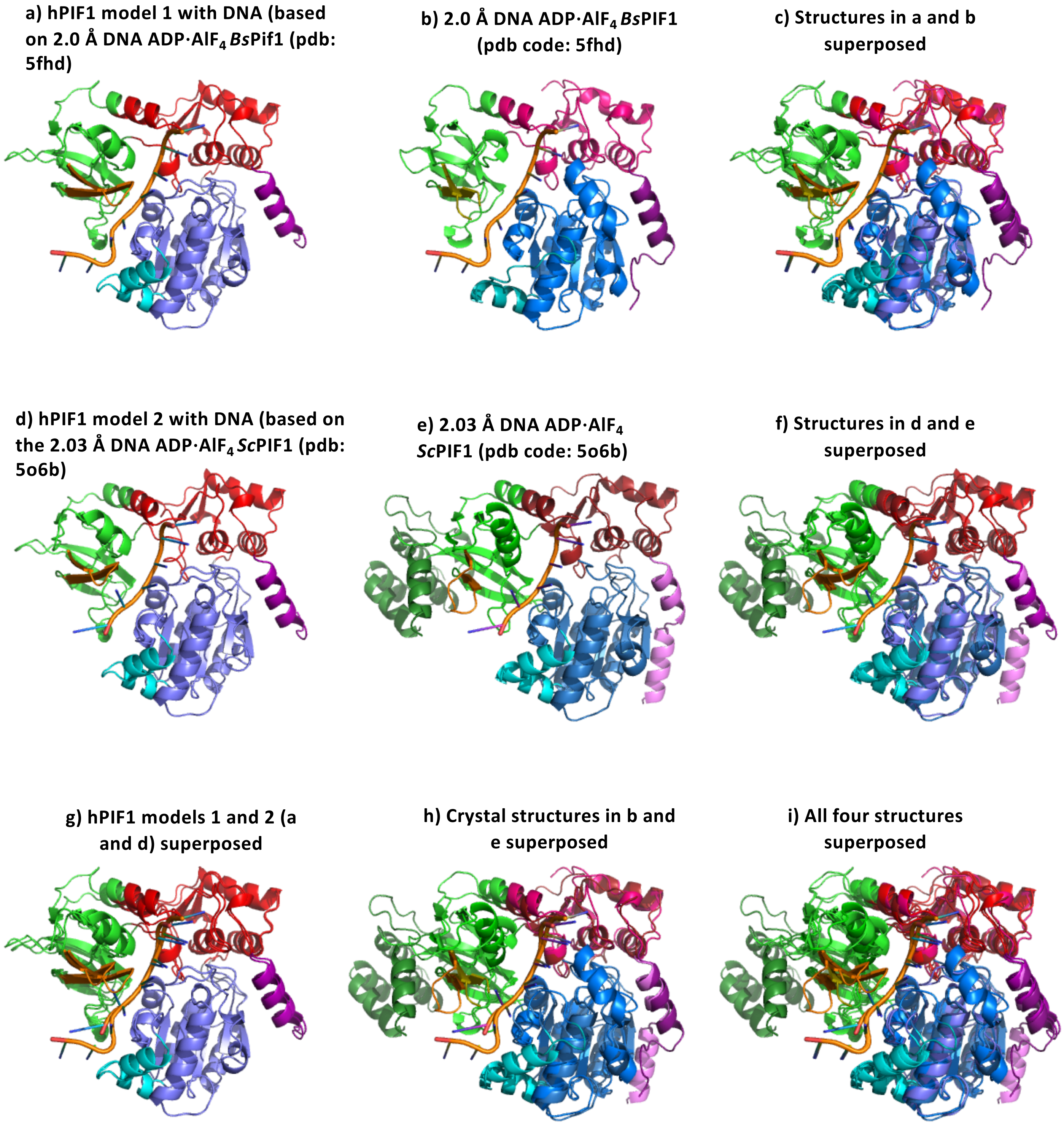

Supplementary Figure S4 – Comparison of models of the DNA bound conformation of human PIF1. Structures are shown in cartoon view – with DNA backbone as yellow tube with bases as lines.

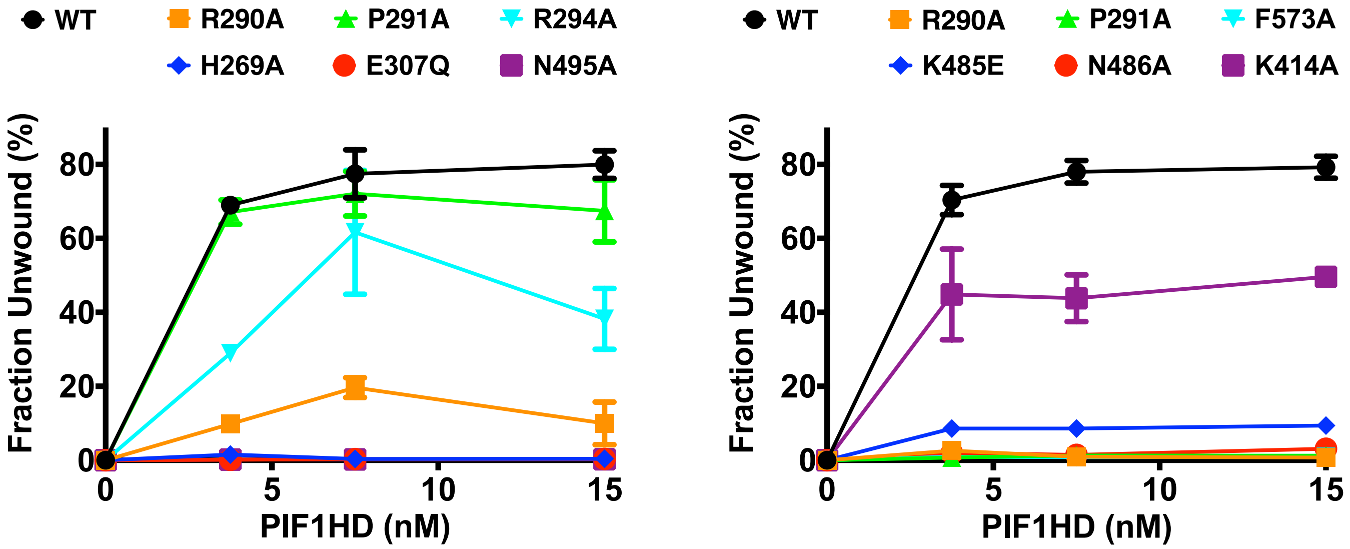

Supplementary Figure S5. Helicase activity of hPIF1 and variant protein forms. Quantified data for all titration points (0.1 nM substrate, 3.75, 7.5 and 15 nM hPIF1) shown in Figure 7, main manuscript. n=4 experimental repeats, mean and standard deviation delimited by the error bars.

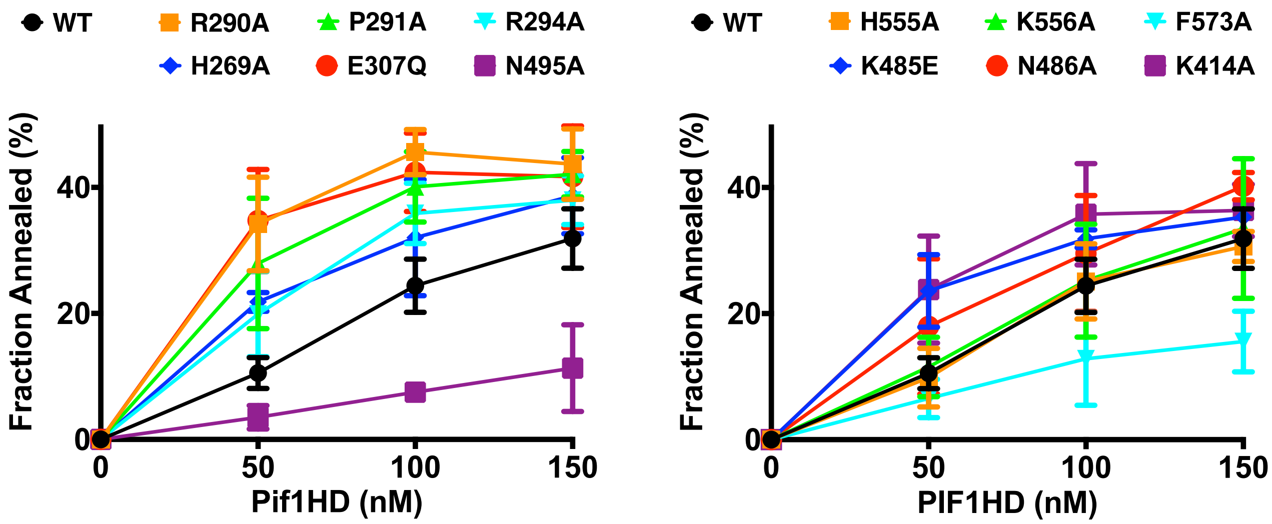

Supplementary Figure S6. DNA strand annealing activity of hPIF1 and variant protein forms. Quantified data for all titration points (0.1 nM substrate, 50, 100 and 150 nM hPIF1) shown in Figure 8, main manuscript. n=4 experimental repeats, mean and standard deviation delimited by the error bars

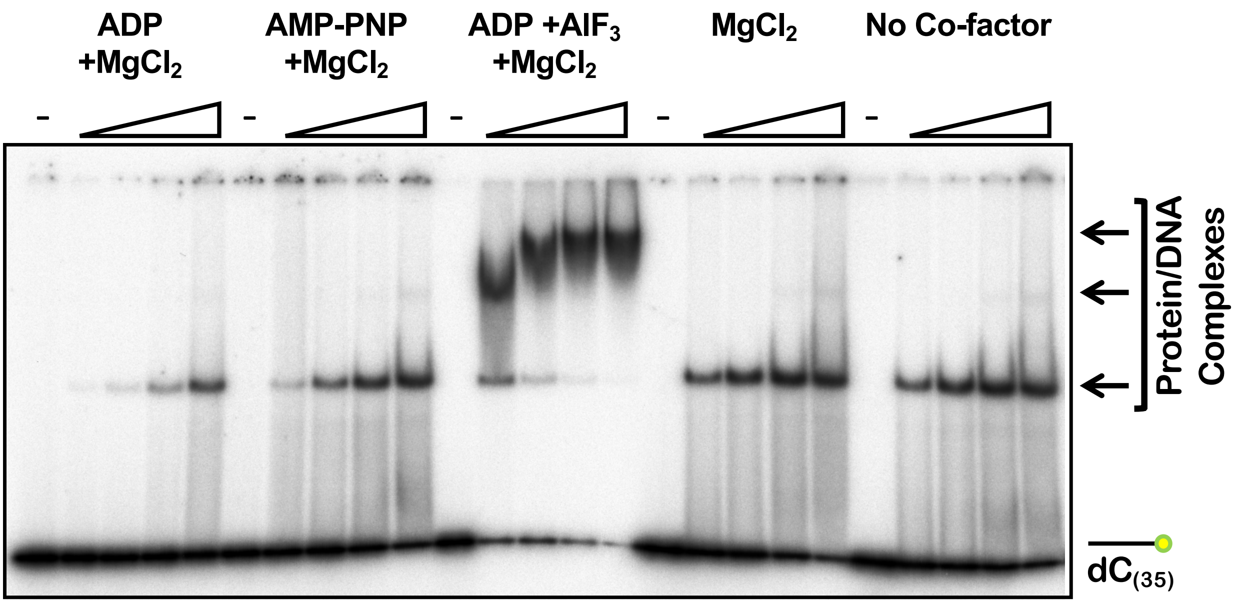

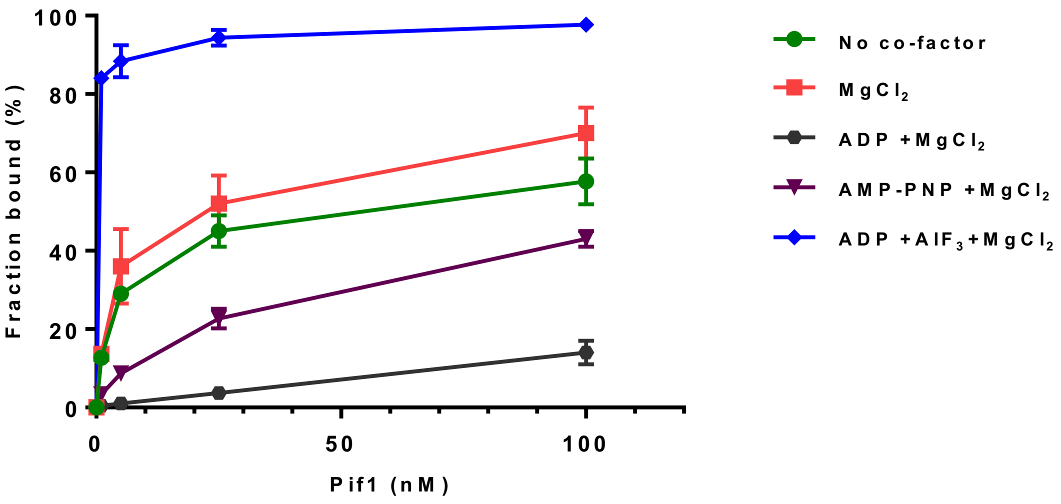

Supplementary Figure S7. hPIF1 binding to ssDNA in the presence and absence of nucleotide cofactors. DNA binding reactions were performed as described in Materials and Methods, main manuscript (0.4 nM ^{32}P=labelled $C_{(30)}$, 1, 5, 25 and 100 nM hPIF, 5 mM nucleotide, 5 mM $MgCl_2$, 5 mM $AlCl_3$ and 37.5 mM NaF). n=3, mean and standard deviation.

	$hPIF1_{AMP\text{-}PNP}$	$hPIF1_{Br}$			$hPIF1_{Apo}$	$hPIF1_{ADF}$
Ligand	AMP-PNP	Br, AMP-PNP			SO_4^{2-}	$ADP\text{-}AlF_4$
Sequence fragment	206-620	206-620			206-641	206-620
Data collection						
X-ray source	I02, DLS	I03, DLS			I04, DLS	I04, DLS
		peak	inflection	remote		
Wavelength, Å	0.97949	0.91966	0.91992	0.91912	0.92819	0.92819
Resolution range, Å	49.95 – 1.13	73.25 – 1.43	76.98 – 1.39	76.94 – 1.87	81.40 – 1.44	104.93 – 3.96
Space group	$C222_1$	$P2_12_12_1$	$P2_12_12_1$	$P2_12_12_1$	$P2_12_12_1$	$P3_121$
Unit-cell parameters, Å	a = 73.3, b = 143.3, c = 77.6	a = 73.3, b = 76.8, c = 142.5	a = 73.2, b = 77.0, c = 142.5	a = 73.2, b = 76.9, c = 142.1	a = 63.5, b = 81.4, c = 91.5	a = b = 209.9, c = 78.9
Number of unique reflections, overall/outer shell[a]	149927 / 9913	148682 / 10867	161273 / 11722	67095 / 4888	86350 / 6315	17633 / 1273
Completeness (%), overall/outer shell[a]	98.7 / 89.63	99.9 / 99.8	99.5 / 98.9	100.0 / 100.0	99.9 / 99.9	100.0 / 100.0
Redundancy, overall/outer shell[a]	6.3 / 4.0	12.8 / 10.7	12.4 / 8.3	12.8 / 13.8	6.6 / 6.8	12.3 / 12.8
I /σ(I), overall/outer shell[a]	11.2 / 1.4	9.6 / 1.0	10.5 / 1.0	5.2 / 1.0	15.9 / 1.3	17.1 / 2.9
R_{merge}[b] (%), overall/outer shell[a]	6.8 / 84.1	14.6 / 231.6	12.5 / 185.1	37.0 / 259.5	4.5 / 132.9	8.0 / 85.7
CC(1/2) (%), overall/outer shell[a]	99.7 / 89.6	99.9 / 59.8	99.9 / 55.2	98.5 / 68.2	99.9 / 51.4	100.0 / 89.2
Refinement and model statistics						
Resolution range (Å)	49.95 – 1.13	71.27– 1.43			60.89 - 1.44	91.03 – 3.96
R-factor[c] (R-free[d]) (%)	12.9 (16.3)	23.3 (27.7)			13.7 (19.5)	17.9 (25.3)
Reflections (working/free)	142572 / 7307	141225/ 7357			81905 / 4365	16607 / 794
Outer shell R-factor[c] (R-free[d]) (%)	30.7 (32.5)	48.5 (51.9)			30.7 (33.9)	33.3 (39.5)
Outer shell reflections (working/free)[g]	9436 / 464	10330 / 532			5981 / 331	1134 / 85
Molecules/asymmetric unit	1	2			1	2
Number of protein non hydrogen atoms	3424	6519			3218	6464
Number of water and small molecules atoms	1754	1260			1002	66
R.m.s. deviation from target[f]						
Bond lengths (Å)	0.029	0.025			0.029	0.010
Bond angles (°)	2.496	2.236			2.452	1.558
Average B-factor ($Å^2$)	21.1	20.2			34.9	151.7
Ramachandran plot[g] (%)	97.9/1.9/0.3	97.4/2.13/0.5			96.8/2.7/0.5	87.8/8.7/3.5

Supplementary Table 1. X-ray Data Collection and Refinement Statistics

[a]The outer shell corresponds to 1.16 – 1.13 Å (hPIF1-AMP-PNP), 1.47 – 1.43 Å (hPIF1-Br peak), 1.43 – 1.39 Å (hPIF1-Br inflection), 1.92 – 1.87 Å (hPIF1-Br remote), 1.48 – 1.44 Å (hPIF1-Apo), 4.06 – 3.96 Å (hPIF1-ADF).

[b]$Rmerge = \sum_{hkl}\sum_i |I_i$ - •*I*• $|/\sum_{hkl}\sum_i$ •*I*• where I_i is the intensity of the *i*th measurement of a reflection with indexes *hkl* and •*I*• is the statistically weighted average reflection intensity.

[c]*R*-factor = $\sum||F_o| - |F_c||/\sum|F_o|$ where F_o and F_c are the observed and calculated structure factor amplitudes, respectively.

[d]*R*-free is the *R*-factor calculated with 5 % of the reflections chosen at random and omitted from refinement.

[e]Outer shell for refinement corresponds to 1.16 – 1.13 Å (hPIF1_AMP-PNP), 1.47 – 1.43 Å (hPIF1_Br peak), 1.43 – 1.39 Å (hPIF1_Br inflection), 1.92 – 1.87 Å (hPIF1-Br remote), 1.48 – 1.44 Å (hPIF1-Apo), 4.06 – 3.96 Å (hPIF1_ADF).

[f]Root-mean-square deviation of bond lengths and bond angles from ideal geometry.

[g]Percentage of residues in most-favoured/ allowed/ outliers regions of the Ramachandran plot.
